# DksA–RNA polymerase interactions support new origin formation and DNA repair in *Escherichia coli*

**DOI:** 10.1111/mmi.14227

**Published:** 2019-03-22

**Authors:** Kamila K. Myka, Kira Küsters, Robert Washburn, Max E. Gottesman

**Affiliations:** ^1^ Department of Microbiology and Immunology Columbia University Medical Center New York NY USA; ^2^ Present address: Molecular Biology Program Memorial Sloan Kettering Cancer Center, Sloan Kettering Institute New York NY USA; ^3^ Present address: Faculty of Biology Westfälische Wilhelms‐Universität Münster Münster Germany

## Abstract

The formation of new replication origins (cSDR) and repair of DNA double‐strand breaks (DSBs) in ***E. coli*** share a commonality. We find that the two processes require the RNAP‐associated factor, DksA. However, whereas cSDR also relies on (p)ppGpp, the alarmone molecule is dispensable for the repair of topoisomerase type II (Top II) DNA adducts and associated DSBs. The requirement for DksA in repair of nalidixic acid (Nal)‐induced DSBs or for the formation of new origins is not suppressed by a ***greA*** deletion mutation, indicating an active role of DksA rather than competition with GreA for insertion into the RNAP secondary channel. Like ***dksA ***mutations, transcription termination factor Rho mutations also confer sensitivity to Nal. The ***rho*** and ***dksA*** mutations are not epistatic, suggesting they involve different repair pathways. The roles of DksA in DSB repair and cSDR differ; certain DksA and RNAP mutants are able to support the first process, but not the latter. We suggest that new origin formation and DNA repair of protein adducts with DSBs may both involve the removal of RNAP without destruction of the RNA:DNA hybrid.

**Abbreviations**
cSDR: constitutive stable DNA replication
RNAP: RNA polymerase
(p)ppGpp: guanosine penta‐ and tetraphosphate
DSB: double‐strand break
dNTPs: deoxyribonucleotide triphosphates
TEC: transcription elongation complex
Nal: nalidixic acid
Top II: type II topoisomerase

## Introduction

*Escherichia coli* utilizes at least three different modes of chromosome replication initiation. In addition to DnaA‐dependent DNA unwinding at *oriC*, replication can initiate at D‐loops and R‐loops (reviewed in Kogoma (1997)). R‐loops consist of RNA insertions into the DNA double helix, generating a RNA:DNA hybrid and a single‐stranded DNA loop. Replication initiation at R‐loops (constitutive stable DNA replication; cSDR) occurs in cells lacking ribonuclease HI (RNase HI), which degrades R‐loop RNA (Ogawa *et al.*, 1984; Maduike *et al.*, 2014). Mutations in RNase HI suppress *dnaA* ^ts^ mutations and enable the growth of *oriC* mutants (Kogoma and von Meyenburg, 1983). RecA is required in cSDR to either form or stabilize R*‐*loops (Kasahara *et al.*, 2000). The RNA within the R‐loop is elongated by DNA polymerase I (Pol I), and subsequent loading of two diverging replisomes by the replication restart machinery creates a new origin of replication (Kogoma and Maldonado, 1997). Replisome reloading proteins PriA and PriB are indispensable for cSDR (Masai *et al.*, 1994; Sandler, 2005). cSDR is also dependent on transcription (von Meyenburg *et al.*, 1987), but the role of the factors associated with RNA polymerase (RNAP) in this reaction has not been studied before. Here we find that DksA, a small RNAP‐binding protein, is required for the formation of new origins in cSDR.

The 17.5 kDa DksA protein shares structural similarity with the two anti‐backtracking factors, GreA and GreB (Perederina *et al.*, 2004). DksA is composed of a globular domain with a zinc‐binding region, a C‐terminal (CT) helix and a coiled‐coil domain that inserts into the secondary channel of RNAP (Perederina *et al.*, 2004; Molodtsov *et al.*, 2018). Unlike the Gre factors, DksA does not induce intrinsic RNA cleavage activity of RNAP (Perederina *et al.*, 2004). DksA, together with the β′ subunit of RNAP, forms (p)ppGpp (guanosine penta‐ and tetraphosphate) binding site 2 (Ross *et al.*, 2016). (p)ppGpp also binds to site 1, located 60 Å away from site 2, on the interface of the ω and β′ RNAP subunits (Ross *et al.*, 2013). (p)ppGpp increases the affinity of DksA to RNAP (Molodtsov *et al.*, 2018). Moreover, the conformation of both DksA and RNAP in the complex changes if (p)ppGpp is present. In the absence of (p)ppGpp, binding of DksA to RNAP bends the β′ rim helix and shifts the βlobe/i4 domain, which is a part of the pincers of the DNA‐binding main channel. This shift might weaken the grip on the non‐template DNA in the transcription bubble and decrease open complex stability. Upon binding of (p)ppGpp to the RNAP–DksA complex, the RNAP is restored to the original apo‐form state. Similarly, DksA conformation reverts to its unbound state in the ternary complex.

DksA, together with (p)ppGpp, participates in the stringent response, a reprogramming of cell metabolism in reaction to environmental stressors, such as nutrient deprivation and heat shock (reviewed in Gaca *et al. *(2015) and Hauryliuk *et al. *(2015)). Overall, the stringent response leads to the repression of genes required for rapid growth (such as rRNA and ribosomal protein genes) and the activation of genes involved in amino acid biosynthesis, nutrient acquisition and stress survival. Cellular DksA concentrations are constant under various growth conditions (Paul *et al.*, 2004; Rutherford *et al.*, 2007). DksA acts both on its own and together with (p)ppGpp to regulate transcription initiation (Paul *et al.*, 2004; Paul *et al.*, 2005) and elongation (Tehranchi *et al.*, 2010; Furman *et al.*, 2012). DksA also prevents transcription stalling when translation and transcription are uncoupled (Zhang *et al.*, 2014) and improves transcription fidelity (Roghanian *et al.*, 2015; Satory *et al.*, 2015). For a recent review describing transcriptional responses to DksA and (p)ppGpp see Gourse *et al. *(2018).

Additionally, both DksA and (p)ppGpp have been implicated in DNA repair. *dksA* mutants are sensitive to DNA damaging agents, such as UV light, the chemotherapeutic agent mitomycin C and nalidixic acid (Nal, an antibiotic belonging to the quinolone drug class) (Meddows *et al.*, 2005; Trautinger *et al.*, 2005). Similarly, the lack of (p)ppGpp sensitized, whereas increased (p)ppGpp levels provided resistance to several genotoxic agents, such as UV, methyl methanesulphonate (MMS), nitrofurazone (NFZ) and 4‐nitroquinoline‐1‐oxide (4NQO) (McGlynn and Lloyd, 2000; Trautinger *et al.*, 2005; Madison *et al.*, 2014; Kamarthapu *et al.*, 2016). (p)ppGpp was suggested to minimize stalled RNAPs, blocking replication fork progression and promote survival *via* a mechanism that involves RecA loading on ssDNA and subsequent SOS induction (McGlynn and Lloyd, 2000; Trautinger *et al.*, 2005). More recently, (p)ppGpp was implicated in the Mfd‐independent transcription‐coupled repair (TCR) pathway, facilitating DNA repair by promoting UvrD‐mediated RNAP backtracking (Kamarthapu *et al.*, 2016). A contradicting study, however, demonstrates that genome‐wide TCR is dependent on Mfd but does not require (p)ppGpp (Adebali *et al.*, 2017).

In this study, we focused on Nal‐induced damage, which introduces both DNA–protein adducts and double‐strand breaks (DSBs). In Gram‐negative bacteria, Nal predominantly targets gyrase, a type II topoisomerase. Gyrase introduces negative supercoils into DNA to relieve torsional stress in front of replisomes and transcribing RNAPs (reviewed in Drlica *et al. *(2008) and Aldred *et al. *(2014)). Type II topoisomerases induce staggered DNA nicks 4 bp apart on both strands and bind covalently to the 5′ phosphate of the two strands, allowing a second DNA duplex to pass through the DSB. Nal stabilizes the transient gyrase–DNA cleavage complex, preventing DNA religation. The gyrase adduct and the DSB pose a barrier to replication and transcription, which leads to irreversible chromosome fragmentation and cell death (Malik *et al.*, 2006). DksA was proposed to enhance the survival after Nal treatment by destabilizing the transcription complexes, thus clearing the way for recombination and DNA repair (Meddows *et al.*, 2005). Nevertheless, a direct role of DksA in the repair of DSBs or the removal of RNAP has not been shown. Additionally, inactive transcription complexes are removed by Rho helicase, which supports chromosome integrity by suppressing replication fork collisions with stalled RNAPs and subsequent formation of DSBs (Washburn and Gottesman, 2011).

Here we describe an interaction of *E. coli* DksA with RNAP that creates new replication origins and promotes the repair of Nal‐induced DSBs*.* We analyzed the role of DksA in *E. coli* MDS42, an MG1655 derivative lacking ~14% of chromosomal DNA, including non‐essential genes and horizontally acquired sequences (Pósfai *et al.*, 2006). We chose this synthetic *E. coli* strain to ensure that the observed cellular responses in the absence of transcriptional factor DksA do not stem from the presence of cryptic prophages. It was shown previously that *rac* prophage present in the MG1655 genome renders it more sensitive than MDS42 to bicyclomycin, an antibiotic targeting transcription termination factor Rho (Cardinale *et al.*, 2008). However, we also tested MG1655 in several experiments.

We find that DksA plays an active role in cSDR and confirm its essentiality in the repair of Nal‐induced DNA damage (Meddows *et al.*, 2005). We assess the roles of other RNAP interacting factors in these DksA‐requiring pathways. Importantly, and in contrast to the repair of phleomycin‐induced DNA lesions, we show that DksA does not act passively to exclude GreA/B from the RNAP secondary channel (Sivaramakrishnan *et al.*, 2017). We propose instead that DksA destabilizes transcription elongation complexes during cSDR and DNA repair but leaves the RNA:DNA hybrid to serve as a primer for new DNA synthesis.

## Results

### DksA is required for cSDR and repair of DNA DSBs bearing protein adducts

**Figure 1:**
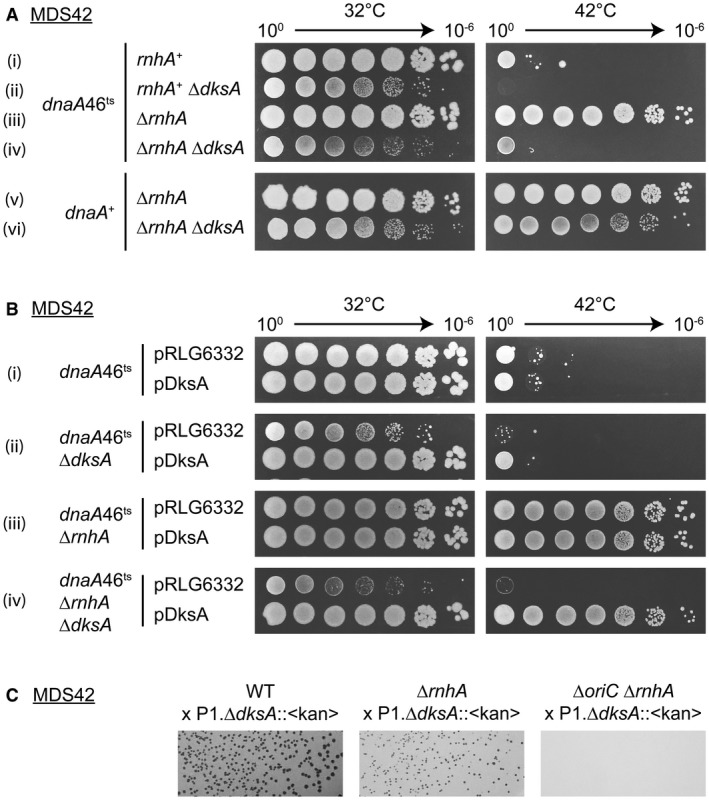
DksA is necessary for *oriC*‐independent replication. A. Deletion of *dksA* prevents cSDR and thus growth at 42°C. The strains tested are (i–vi): KM644, KM805, KM650, KM883, 10562, KM777. B. Growth is restored by the expression of a plasmid‐encoded DksA protein. Strains (i–iv): KM644, KM803, KM650, KM882; pDksA = pRLG6333. C. *dksA* cannot be deleted from a Δ*oriC* Δ*rnhA* strain. Strains (left to right): MDS42, 10562, RSW764. P1 was made on JW0141, a Keio collection Δ*dksA*::kan.

In the absence of RNase HI (Δ*rnhA*), *E. coli* cells are capable of replicating using not only the chromosomal origin of replication *oriC*, but also DnaA‐independent *oriK* sequences, which fire randomly with respect to the cell cycle (von Meyenburg *et al.*, 1987; Maduike *et al.*, 2014). *oriK *sites contain R‐loops that are extended by DNA Pol I to form new replication origins. This pathway, named cSDR, enables a strain with a temperature‐sensitive DnaA protein to grow at non‐permissive temperatures. We introduced the *dnaA*46^ts^ and Δ*rnhA* mutations into *E. coli* MDS42 (Fig. 1A). A *dnaA*46^ts^ mutant cannot grow at 42°C, whereas a *dnaA*46^ts^ Δ*rnhA* grows at the highest dilution tested (Fig. 1A i and iii). Since the proteins that extend an RNA primer with dNTPs and reload a replisome have been studied previously, we decided to focus on the possible role of RNAP in the formation of new origins. We studied several RNAP mutants and factors that interact with RNAP, such as transcription factor DksA, anti‐backtracking Gre factors and (p)ppGpp. Fig. 1A shows that the deletion of *dksA* prevents the growth of *dnaA*46^ts^ Δ*rnhA* at 42°C (Fig. 1A iv). DksA expressed from a plasmid reversed this phenotype (Fig. 1B iv). A *dnaA* ^+^ Δ*rnhA* Δ*dksA *mutant grew at 42°C, supporting a direct role for DksA in cSDR (Fig. 1A vi). In contrast to WT or Δ*rnhA* strains, we were unable to introduce Δ*dksA *into a Δ*oriC *Δ*rnhA* strain, which replicates only *via* cSDR (Fig. 1C). The requirement for DksA in cSDR is not specific to MDS42 background, as the deletion of *dksA *in MG1655 *dnaA*46^ts^ Δ*rnhA* strain also blocked the growth at 42°C (Fig. 7 v and vii). These data indicate that DksA enables the formation of new *E. coli* origins.

**Figure 2:**
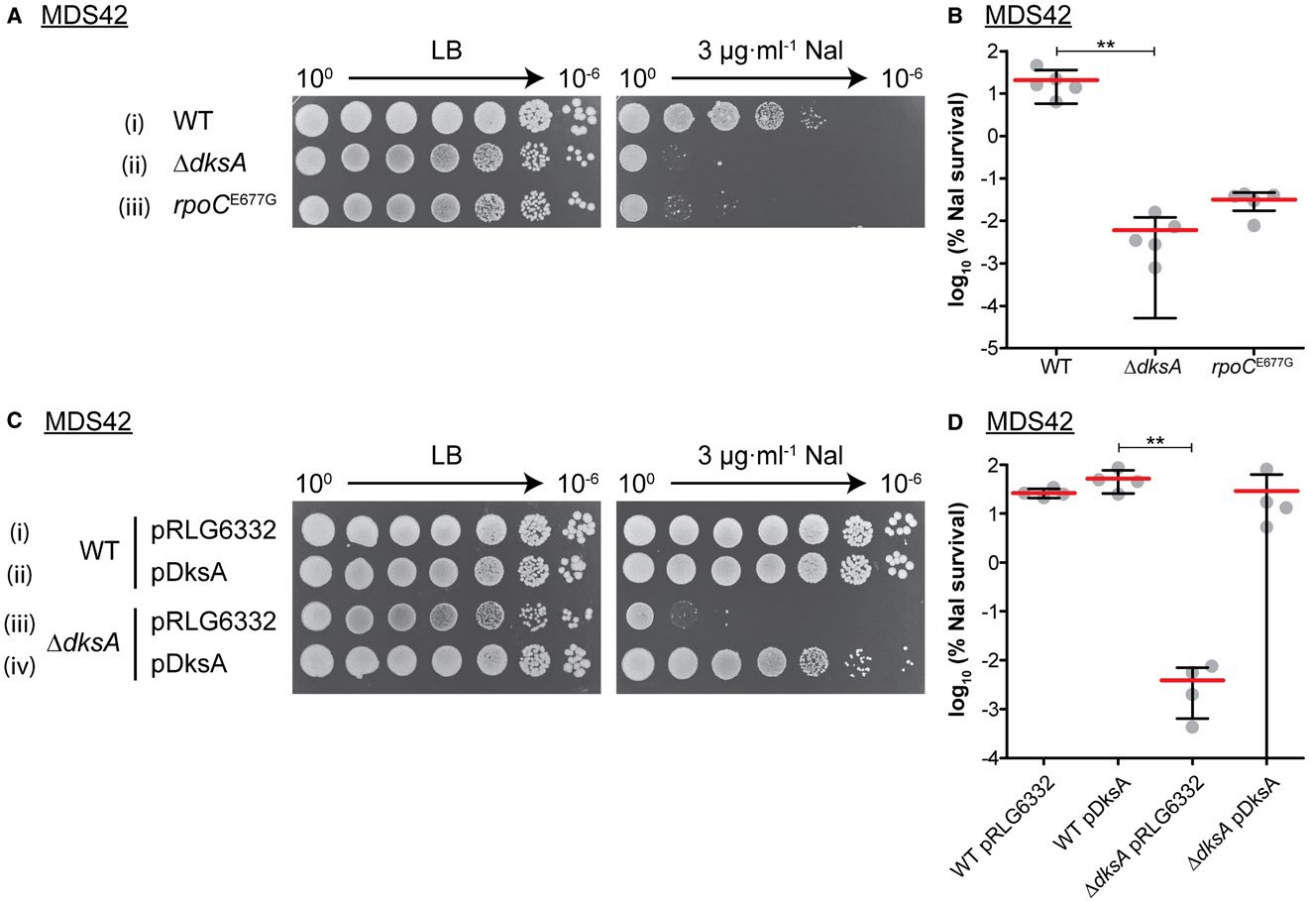
Interaction between RNAP and DksA is required for DNA repair. A. Both Δ*dksA *and a ‘DksA‐blind’ *rpoC* ^E677G^ mutant are sensitive to Nal. Strains (i–iii): MDS42, KM885, KM807. B. Calculated percentage survival of strains on LB + Nal vs. LB alone. Graph shows mean percentage survival with one standard deviation. Statistical analysis was performed using a nonparametric Kruskal–Wallis test with Dunn’s post test, comparing all data sets. **p < 0.01, *n* = 5. C. Nalidixic acid sensitivity of Δ*dksA* is suppressed by the expression of plasmid‐encoded DksA. Strains (i–ii): MDS42, (iii–iv): KM885. pDksA = pRLG6333. D. As in (B), *n* = 4. [Colour figure can be viewed at wileyonlinelibrary.com].

It has been shown that* dksA *mutants are sensitive to nalidixic acid (Meddows *et al.*, 2005). Nal inhibits the bacterial DNA gyrase A subunit, creating a DNA DSB with stable 5′ DNA gyrase adducts. This structure creates a lethal barrier for replication and transcription. At present, the repair of Nal lesions is not fully understood. We hypothesized that the repair of Nal‐induced DSBs could require the interaction of DksA with RNAP to create an RNA primer for DNA synthesis, as is the case in cSDR. We decided to compare the requirements for the formation of new origins (cSDR) and DSB repair. We confirmed the sensitivity of Δ*dksA* mutants to Nal (Fig. 2A ii and B) and showed complementation by plasmid‐encoded DksA (Fig. 2C iv and D).

### The ‘DksA‐blind’ RNAP mutant does not support oriC‐independent replication or DSB repair

**Figure 3:**
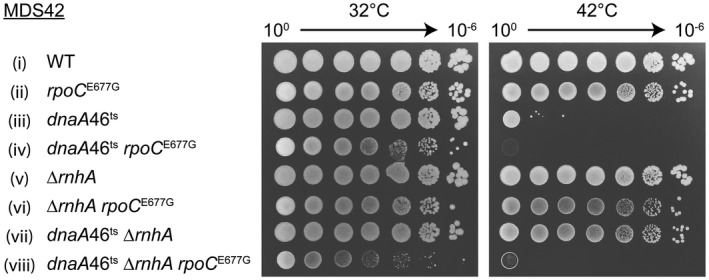
Interaction between DksA protein and RNAP is required for cSDR. A ‘DksA‐blind’ *rpoC* ^E677G^ mutant cannot replicate *via* cSDR. Strains (i–viii): MDS42, KM807, KM644, KM809, 10562, KM829, KM650, KM801.

To confirm that the role of DksA in cSDR and DSB repair involves its interaction with RNAP, we tested a ‘DksA‐blind’ RNAP mutant, *rpoC* ^E677G^ that does not bind to DksA (Satory *et al.*, 2013; Ross *et al.*, 2016). The triple mutant *dnaA*46^ts^ Δ*rnhA* *rpoC* ^E677G^ was unable to grow at the non‐permissive temperature, unlike the parental *dnaA*46^ts^ Δ*rnhA* (Fig. 3 viii and vii). Control *dnaA^+^* strains displayed a similar colony forming ability at the permissive and non‐permissive temperatures (Fig. 3 i–ii, v–vi). *rpoC* ^E677G^ mutant was also sensitive to Nal (Fig. 2A iii and B). We conclude that the interaction between DksA and RNAP is required for both cSDR and DNA repair.

### (p)ppGpp is required for cSDR but not for the repair of Nal‐induced DNA damage

**Figure 4:**
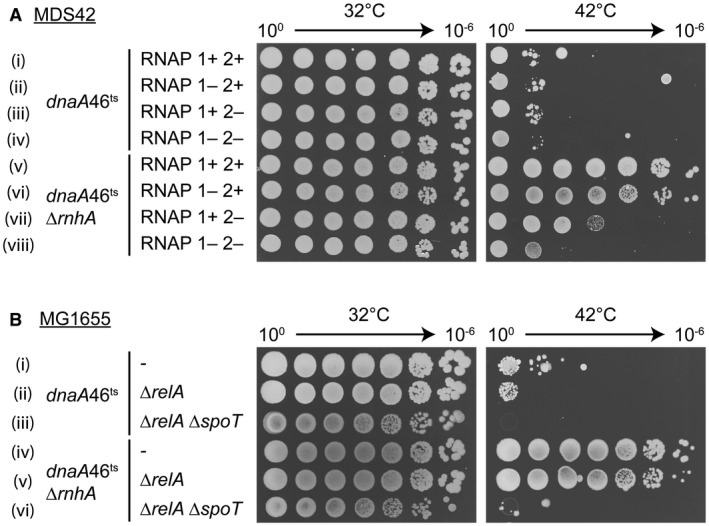
Mutations in RNAP (p)ppGpp‐binding sites and the absence of (p)ppGpp inhibit cSDR. A. RNAP (p)ppGpp‐binding site 2 plays a major role in cSDR in MDS42. Strains (i–viii): KK06A, KM899, KK08A, KM911, KK07B, KM901, KK09A, KM913. B. (p)ppGpp is required for *oriC*‐independent replication. Strains (i–vi): KM712, KM1136, KM1137, KM1171, KM1173, KM1236.

DksA often acts in concert with (p)ppGpp to regulate transcription initiation. We asked if DksA requires (p)ppGpp to promote new origin formation. First, we deleted *relA*, which encodes the major (p)ppGpp synthetase in *E. coli*. The *dnaA*46^ts^ Δ*rnhA* Δ*relA *strain was able to grow at both low and high temperatures, although colony size at 42°C was decreased relative to the *relA* ^+^ parent (Fig. S1A). Attempts to additionally delete *spoT* (gene encoding a bifunctional (p)ppGpp synthase/hydrolase) and create a (p)ppGpp^0^ MDS42 strain were unsuccessful. In *E. coli, *(p)ppGpp binds to RNAP and also to other protein targets. To determine if the interaction of (p)ppGpp and RNAP is required for cSDR, we investigated RNAP polymerase mutants defective in (p)ppGpp binding. *E. coli *RNAP carries two (p)ppGpp binding sites. Site 1 is formed by the ω and β′ subunits, whereas site 2 is formed by DksA and β′. The mutations disrupting site 1 (RNAP 1‐) include a deletion of several amino acids from the ω subunit (*rpoZ* ^Δ2–5^) and three point mutations in the β′ subunit (*rpoC* ^R362A R417A K615A^) (Ross *et al.*, 2013). The mutations disrupting site 2 (RNAP 2‐) are limited to two substitutions in the β′ subunit (*rpoC* ^N680A K681A^) that prevent (p)ppGpp but not DksA binding (Ross *et al.*, 2016). We introduced the RNAP 1‐ and 2‐ mutations into the *dnaA*46^ts^ Δ*rnhA* strain and tested growth at high temperatures. Mutations in either or both of (p)ppGpp‐binding sites did not affect the growth of the *dnaA*46^ts^ strains at 32°C (Fig. 4A i–iv). At the non‐permissive temperature, mutations in RNAP binding site 1 reduced colony size, but did not prevent colony formation by *dnaA*46^ts^ Δ*rnhA* (Fig. 4A vi). In contrast, site 2 mutations inhibited the growth of *dnaA*46^ts^ Δ*rnhA *at 42°C approximately 1000‐fold (Fig. 4A vii). Deletion of both (p)ppGpp‐binding sites was even more inhibitory on the growth of *dnaA*46^ts^ Δ*rnhA *at 42°C (Fig. 4A viii).

**Figure 5:**
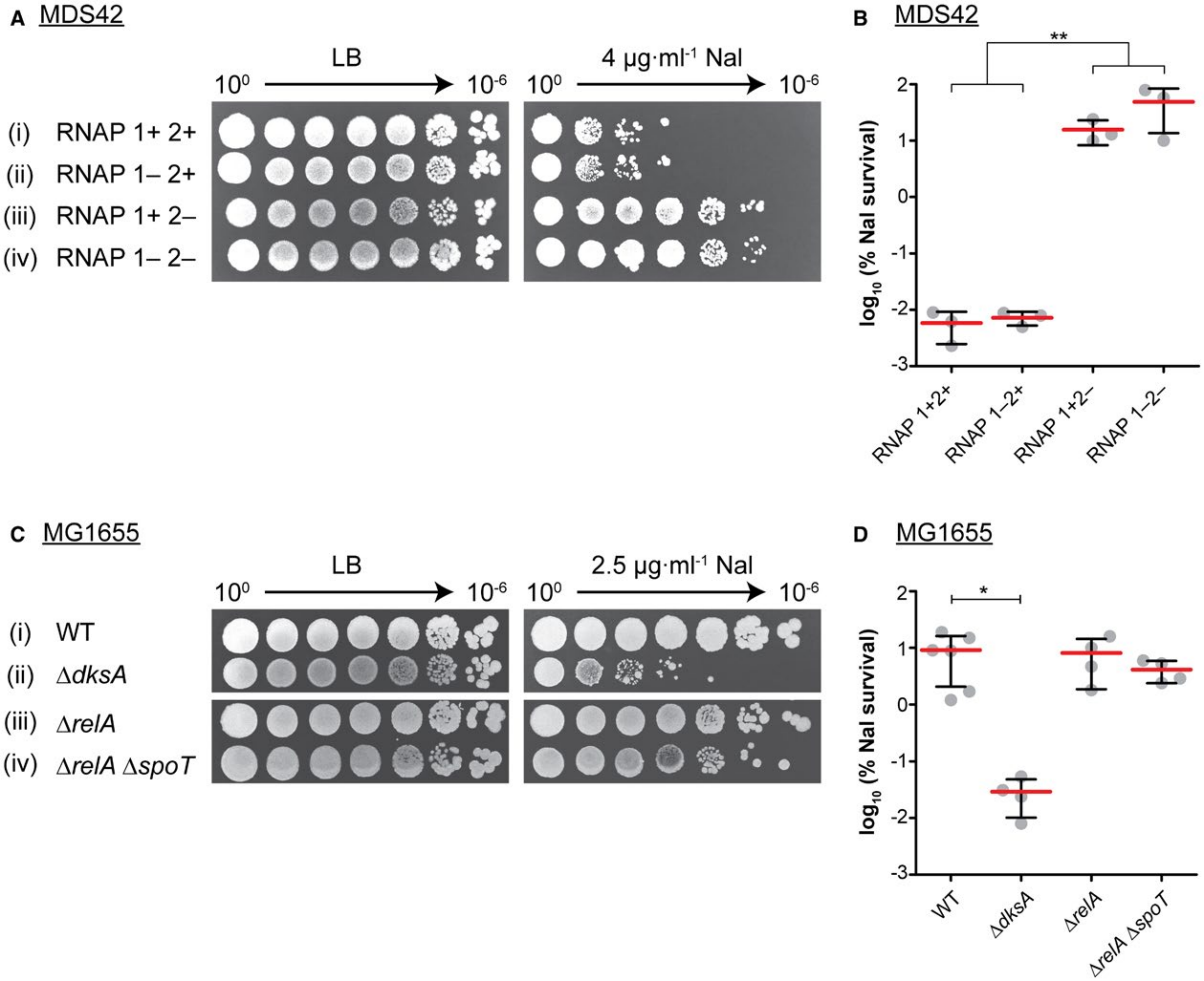
The RNAP (p)ppGpp‐binding site 2 mutation enhances resistance to nalidixic acid, whereas the absence of (p)ppGpp fractionally decreases it. A. The RNAP site 1 mutation did not affect growth on Nal, whereas the RNAP site 2 mutant was more resistant than the wild type. Nalidixic acid concentration was increased to 4 µg ml^–1^. Strains (i–iv): KK04A, KM915, KK05A, KM917. B. Calculated percentage survival of strains on LB + Nal vs. LB alone. Graph shows mean percentage survival with one standard deviation. Statistical analysis was performed using a nonparametric two‐tailed Mann–Whitney test, comparing combined data for RNAP 2 + vs. RNAP 2‐ mutants. **p < 0.01, *n* = 3. C. (p)ppGpp is not required for the repair of nalidixic acid‐induced DNA damage. The Nal concentration was decreased to 2.5 µg ml^−1^ due to higher sensitivity of MG1655‐derived strains. Strains (i*–*iv): MG1655, KM773, RLG850, RLG847. D. As in (B) but statistical analysis was performed using a nonparametric Kruskal–Wallis test with Dunn’s post test, comparing all data sets. *p < 0.05, *n* = 6, 4, 4, 4, respectively. [Colour figure can be viewed at wileyonlinelibrary.com].

In contrast, deletion of (p)ppGpp‐binding sites had an opposite effect on the sensitivity of strains to Nal. The strains lacking RNAP site 1 grew comparably to the parent, whereas the growth of the RNAP site 2 mutant and the RNAP sites 1 and 2 mutant was significantly improved relative to the wild type (Fig. 5A and B; Fig. S2A and B). Although increased resistance of RNAP 2‐ to Nal was true for both MDS42 and MG1655 background (Figs 5 and S2), the effect of mutations in (p)ppGpp‐binding sites 1 and 2 in cSDR appears to be different depending on the strain background. In MDS42, site 2 plays a bigger role, whereas in MG1655, site 1 seems more important (Fig. S1B). We do not yet have an explanation for this phenomenon.

To eliminate the possibility that the RNAP mutations themselves, rather than the lack of interaction with (p)ppGpp affect R‐loop‐initiated replication and DSB repair, we utilized the MG1655 background. Here, we were able to construct a *dnaA*46^ts^ Δ*rnhA *(p)ppGpp^0^ strain lacking both *relA* and *spoT* and test it for growth at permissive and non‐permissive temperatures. We found that the lack of (p)ppGpp prevented cSDR in MG1655 (Fig. 4B vi) to a larger extent than mutations in the (p)ppGpp‐binding sites 1 and 2 (Fig. S1B). However, while RNAP site 2 mutation significantly increased MG1655 resistance to Nal (Fig. S2A and B), the (p)ppGpp^0^ mutant was ~10‐fold more sensitive to Nal than the parental strain (Fig. 5C and D; Fig. S2C). The discrepancy between the (p)ppGpp^0^ and RNAP 1‐2‐ phenotypes suggests that the RNAP mutations *per se* increase resistance to Nal. We confirmed all the (p)ppGpp^0^ phenotypes by showing that strains failed to grow on a minimal medium and, therefore, had not accumulated suppressors (Figs S1C and S2D). We conclude that (p)ppGpp plays a significant role in cSDR but not in the repair of Nal‐induced DNA damage.

### Anti‐backtracking factors are not essential for cSDR

**Figure 6:**
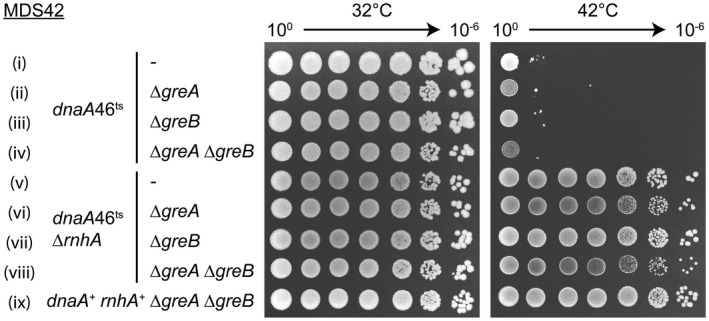
GreA and GreB anti‐backtracking factors are not required for cSDR. Deletion of *greA* or *greB* does not prevent cSDR in the *dnaA*46^ts^ Δ*rnhA* strain. Strains (i–ix): 10583, 12334, 12336, KM1019, KM554, KM586, KM588, KM1021, KM982.

The coiled‐coil domain of DksA protein inserts itself within the RNAP secondary channel. The anti‐backtracking factors GreA and GreB share a similar structure, enter the secondary channel and compete with DksA for binding to RNAP (Vinella *et al.*, 2012). To examine their potential role in cSDR, we deleted each of the genes from the *dnaA*46^ts^ Δ*rnhA* strain and assayed the growth at 42°C (Fig. 6 vi–vii). Deletion of *greA* or *greB* did not block the growth at non‐permissive temperatures, indicating that the lack of one of the factors does not prevent cSDR. A double *greA greB* deletion mutant is temperature sensitive in *E. coli *MG1655 but not in MDS42 (Fig. 6 ix). We were able, therefore, to construct and assay an MDS42 *dnaA*46^ts^ Δ*rnhA* Δ*greA* Δ*greB* mutant. This strain, which lacks both Gre factors, grows at the non‐permissive temperature (Fig. 6 viii). Moreover, a Δ*oriC* Δ*rnhA* Δ*greA* Δ*greB* mutant was also viable (data not shown). Taken together, the data confirm that the anti‐backtracking factors are not required for cSDR.

### DksA plays an active role in cSDR and DSB repair

**Figure 7:**
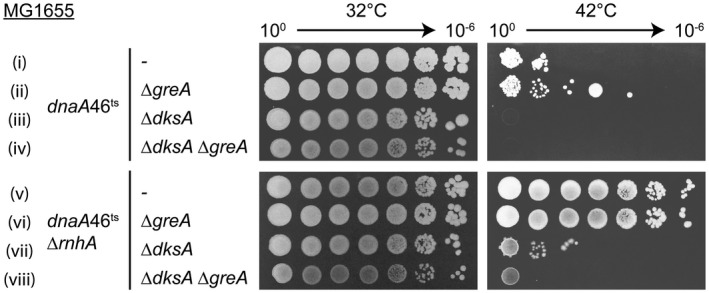
DksA plays an active role in cSDR. Deletion of *greA* did not restore cSDR to the *dnaA*46^ts^ Δ*rnhA* Δ*dksA* mutant. Strains (i–viii): KM727, KM1058, KM1060, KM1066, KM993, KM1062, KM1064, KM1068.

It is possible that GreA/GreB blocks cSDR, and that the role of DksA is to reduce the entry of these factors into the RNAP secondary channel (Sivaramakrishnan *et al.*, 2017). To address this question, we attempted to delete *greA* from *dnaA*46^ts^ Δ*rnhA* Δ*dksA*. We reasoned that if the increased interaction of GreA with RNAP in the absence of DksA inhibited cSDR, then deleting *greA* should restore the ability of the strain to replicate in an *oriC*‐independent manner. We were not able to construct the double Δ*dksA* Δ*greA* mutant in MDS42. We could however construct the *dnaA*46^ts^ Δ*rnhA* Δ*dksA* Δ*greA* mutant in the MG1655 background (Fig. 7). The mutant was unable to form colonies at the non‐permissive temperature, indicating that DksA exclusion of GreA/B does not account for the DksA requirement for *oriC*‐independent replication (Fig. 7 viii).

**Figure 8:**
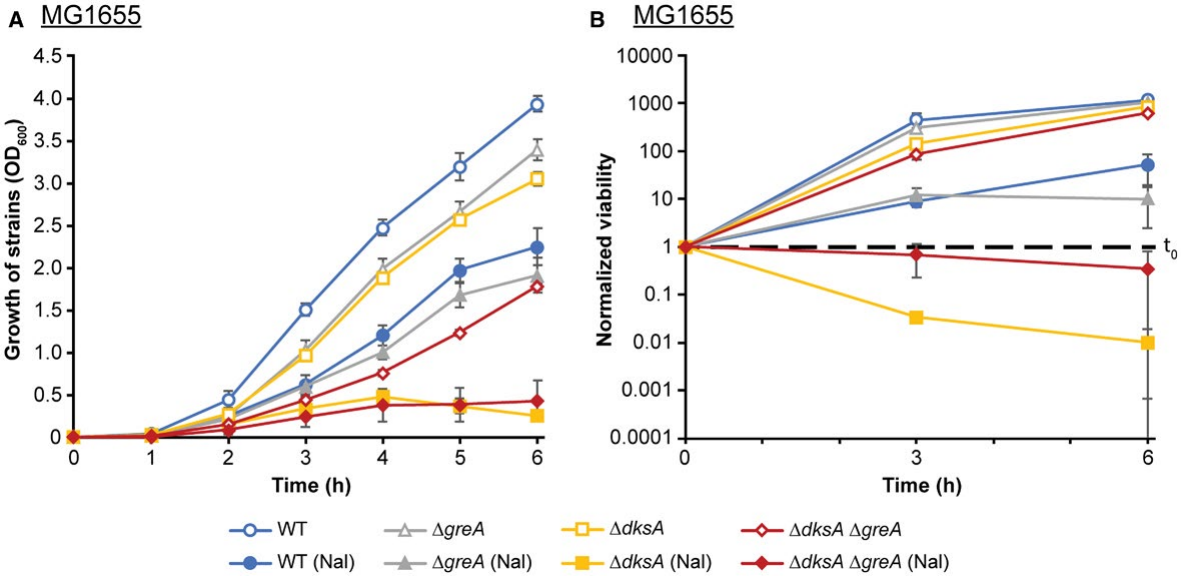
Deletion of *greA* did not suppress Δ*dksA* nalidixic acid sensitivity. A. Growth curve in the absence and presence of Nal. Exponential phase cultures were diluted to OD_600_ = 0.01 and the absorbance was measured hourly. Strains: MG1655, KM1034, KM773, KM1054. B. Quantification of the increase in viable count at 3 and 6 h compared to the viability at *t*_0_ arbitrarily set to 1 for each replicate. Graph represents mean and standard deviation, *n* = 3. Standard deviation value higher than the mean value results in negative error bars crossing the *x*‐axis when the *y*‐axis is in a logarithmic scale.

Next, we asked if the deletion of *greA* rescues the Nal sensitivity of a Δ*dksA* mutant, as would be predicted if DksA acted passively. *dksA* mutants are sensitive to the radiomimetic drug, phleomycin (Sivaramakrishnan *et al.*, 2017) (Fig. S3A). A *greA* deletion not only improved wild‐type growth in phleomycin, but also suppressed the sensitivity of a *dksA* mutant. This suggested that DksA acts passively to enhance DNA damage repair by excluding GreA from the RNAP secondary channel, thus favoring RNAP backtracking (Sivaramakrishnan *et al.*, 2017). We therefore tested the Nal sensitivity of a MG1655 Δ*dksA* Δ*greA *mutant. Initially, we tested the susceptibility to Nal as in previous experiments, by serially diluting strains and plating them on LB agar containing defined Nal concentrations. We observed that Δ*dksA* Δ*greA *formed colonies on higher dilutions than Δ*dksA* alone (Fig. S3B). However, unlike WT or Δ*greA *strains, Δ*dksA* Δ*greA *formed single colonies starting from the 10^−1^ dilution. This suggested to us that Nal is bacteriostatic for the double mutant and prompted us to use an additional assay to investigate the effect of Nal on Δ*dksA* Δ*greA. *Growth in the presence of the antibiotic was monitored by the absorbance of cultures at OD_600_, as well as by counting the viable cells present in the cultures after 3 and 6 h of incubation. The Δ*greA* mutant and the wild‐type strain were equally sensitive to Nal and increased in cell mass as well as viability during growth in LB with similar kinetics (Fig. 8). The Δ*dksA* mutant was very sensitive to Nal showing little increase in culture density and decreasing rapidly in viability with exposure to the inhibitor. After 3 h in Nal, only 3% of the initial Δ*dksA* culture survived, and by 6 h the viability count was 1% that of the input. The double Δ*dksA* Δ*greA *strain was also sensitive to Nal, showing little increase in OD_600_ over the 6 h time period (Fig. 8A). However, Nal was bacteriostatic for the double mutant, rather than bactericidal, indicating that Δ*greA* has some protective effect in a Δ*dksA *mutant (Fig. 8B). These findings indicate that DksA plays an active role in the repair of Nal lesions, rather than the passive one of excluding GreA.

### Rho‐dependent termination is required for the repair of Nal‐induced DNA damage

**Figure 9:**
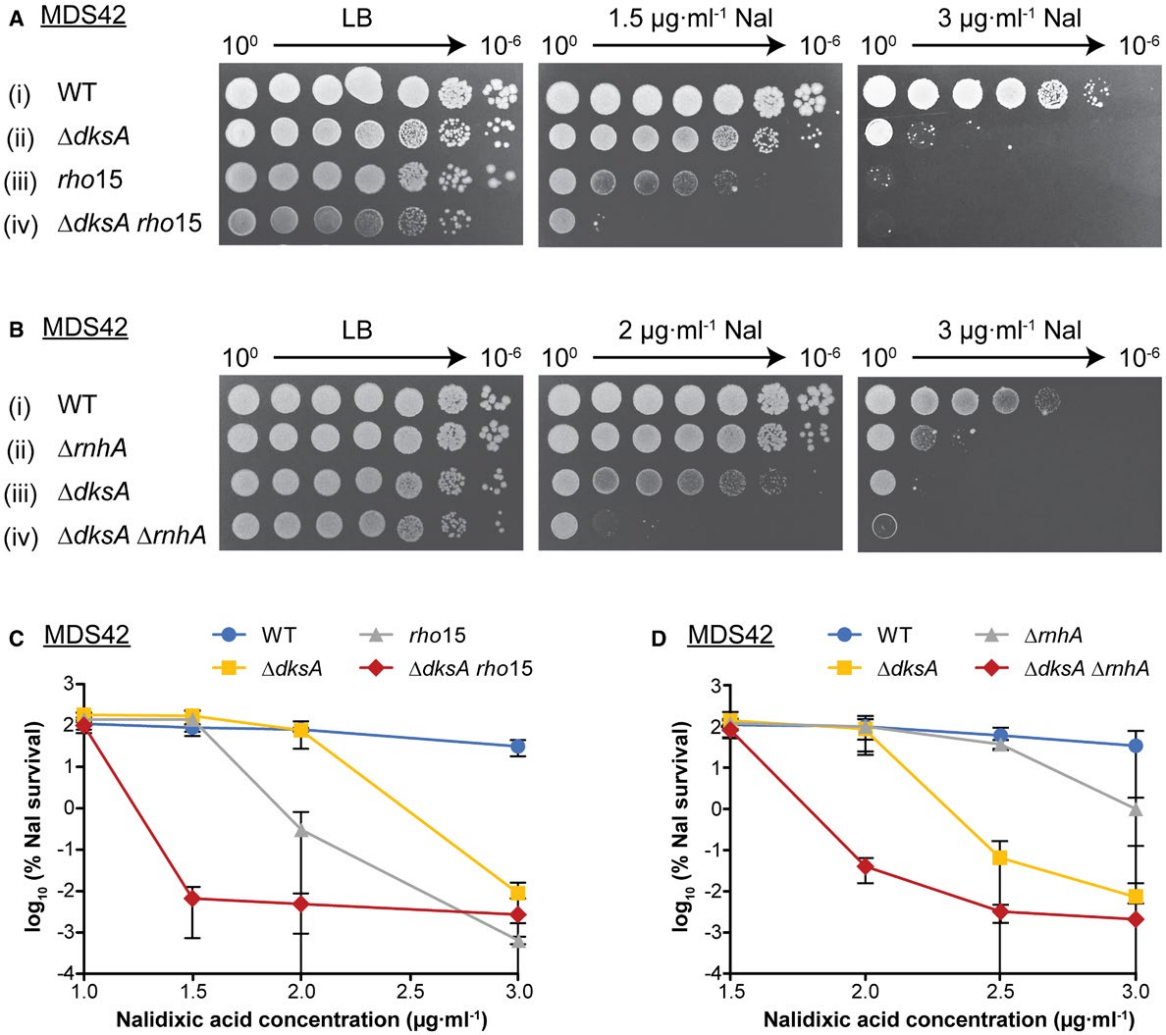
DksA participates in a different DNA repair pathway than Rho and RNase HI. A. The *rho*15 mutant is more sensitive to Nal than Δ*dksA, *and together the mutations have an additive inhibitory effect. Strains (i–iv): MDS42, KM885, 10598, 12478. B. The Δ*rnhA* mutant is more resistant than Δ*dksA *to Nal and they are not epistatic. Strains (i–iv): MDS42, 10562, KM885, KM777. C. Calculated percentage survival of strains on LB + Nal vs. LB alone. Graph shows mean with standard deviation, *n* = 3, 2, 3, 4 for Nal concentration 1, 1.5, 2 and 3 µg ml^−1^, respectively. D. As in (C) n = 3, 3, 3, 6 for Nal concentration 1.5, 2, 2.5 and 3 µg ml^−1^, respectively.

Inhibition of Rho‐dependent transcription termination leads to chromosomal DSBs (Dutta *et al.*, 2011; Washburn and Gottesman, 2011). This is thought to result from transcription–replication clashes, rather than from failure to repair DSBs. We find that Rho is required to repair DSBs induced by Nal and/or to suppress clashes resulting from such breaks. As shown in Fig. 9, the *rho*15 missense mutant is highly sensitive to Nal (Fig. 9A iii and C). To determine if Rho and DksA are part of the same repair pathway, we constructed a strain bearing both the Δ*dksA* and *rho*15 mutations. To test for epistasis, we lowered the Nal concentration from 3 to 1.5 µg ml^−1^. At this concentration, both the Δ*dksA* mutant and the wild type grew (Fig. 9A i–ii), but the *rho*15 mutant was ~100‐fold more sensitive than the parental strain (Fig. 9A iii and i). The double mutant was more growth‐defective than the *rho*15 mutant by itself (Fig. 9A iii–iv). We conclude, therefore, that Rho and DksA are involved in different pathways of recovery from Nal‐induced DNA damage.

### Roles of RNase HI and DksA in Nal‐induced DNA damage repair

Inactivation of RNase HI is necessary for new DNA origin formation from the resulting persistent R‐loops. On the other hand, R‐loops can initiate DNA breaks (Wimberly *et al.*, 2013). To test if the deletion of *rnhA* affects Nal sensitivity and if DksA and RNase HI act in the same pathway, we constructed Δ*rnhA* and Δ*dksA *Δ*rnhA* mutants. Abrogation of RNase HI activity exacerbated Nal sensitivity ~100‐fold at 3 µg ml^−1^ of Nal compared to the wild‐type strain (Fig. 9B ii and D). At lower Nal concentrations, the growth of the *rnhA* mutant was similar to that of the wild type. Combined, the Δ*rnhA* and Δ*dksA* mutations increased Nal sensitivity more than either mutation alone (Fig. 9B and D). We propose that both DksA and RNase HI act to prevent or repair Nal‐induced DNA damage, but that they participate in separate pathways.

### A mutation in the RNAP main channel suppresses Nal sensitivity of the dksA mutant and restores new origin formation

**Figure 10:**
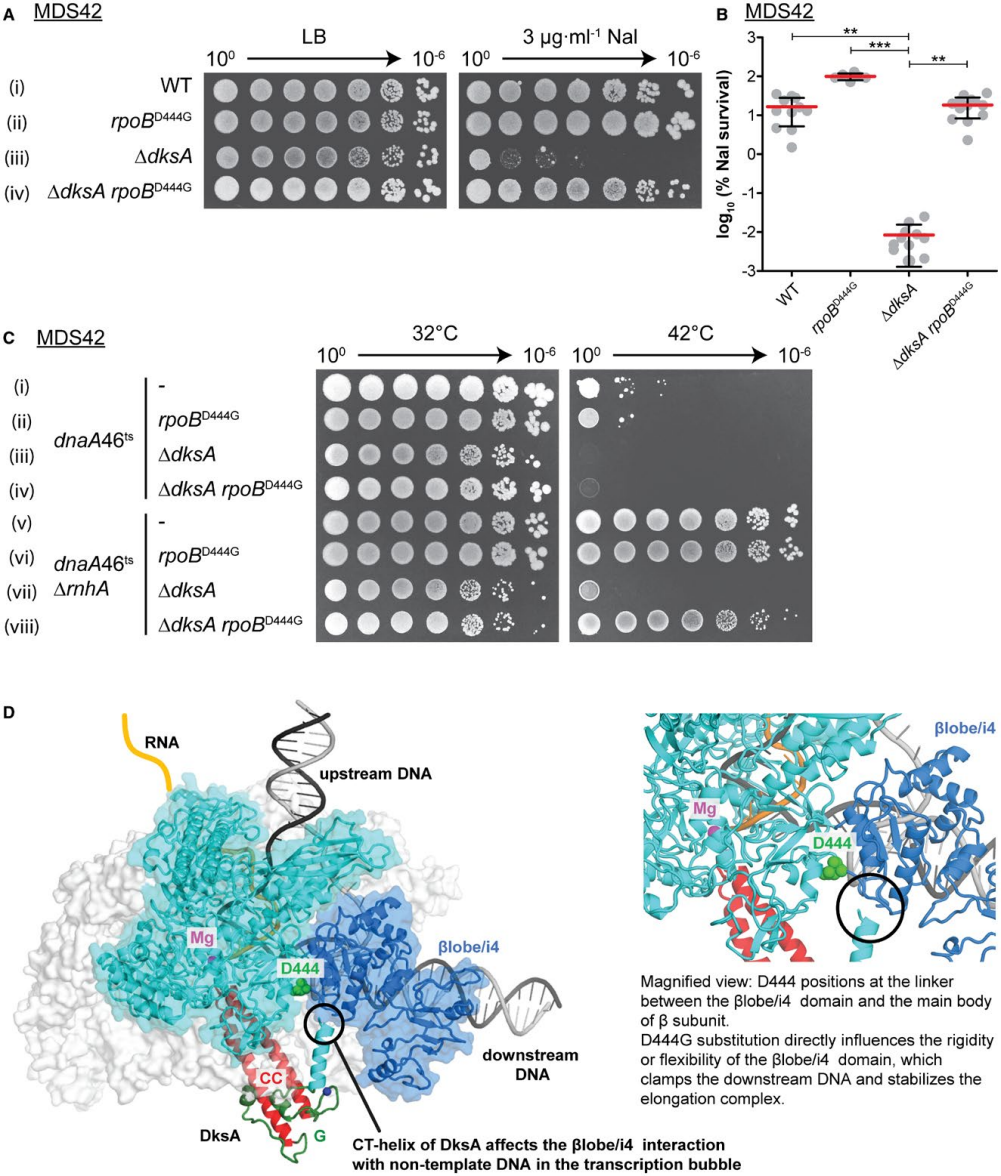
A mutation in the β subunit of RNAP rescues Δ*dksA *sensitivity to nalidixic acid and allows for replication *via* cSDR. A. The *rpoB* ^D444G^ mutation enables Δ*dksA* to grow in the presence of Nal. Strains (i–iv): MDS42, KM1047, KM885, 12481. B. Calculated percentage survival of strains on LB + Nal vs. LB alone. Graph shows the mean percentage survival with one standard deviation. Statistical analysis was performed using a nonparametric Kruskal–Wallis test with Dunn’s post test. **p < 0.01, ***p < 0.001. *n* = 11, 6, 11, 13. C. *rpoB* ^D444G^ enables cSDR in the Δ*dksA* mutant. Strains (i–viii): KM644, KM1009, KM803, KM1013, KM650, KM1011, KM882, KM1017. D. Structural model of RNAP in complex with DksA with RNAP residue β^D444^ annotated.

To test the hypothesis that DksA might decrease the stability of RNAP and thus contribute to cSDR and DNA repair, we tested several previously isolated RNAP mutants that allow replication in the absence of accessory replicative helicases Rep and UvrD (Baharoglu *et al.*, 2010). One such mutation, *rpoB* ^D444G^ efficiently suppressed the Nal sensitivity of the *dksA* mutant (Fig. 10A iii–iv and B). Additionally, as shown in Fig. 10C, the *rpoB* ^D444G^ substitution was also able to restore cSDR in the *dnaA*46^ts^ Δ*rnhA *Δ*dksA* strain (Fig. 10C vii–viii). *rpoB* ^D444G^ was shown not only to bypass the need for accessory replicative helicases required to remove transcribing RNAPs, the major obstacle to replication, but also to improve UV resistance of *ruvABC*, a Holliday junction resolvase mutant (Baharoglu *et al.*, 2010). Based on these observations, it was proposed that the *rpoB* ^D444G^ mutation increases the intrinsic instability of the RNAP–DNA complexes, facilitating both the removal of RNAP upon replication–transcription collisions and replication restart (Baharoglu *et al.*, 2010). Our results are consistent with this model and suggest that destabilization of RNAP is required both for the formation of new origins and for DNA repair.

### Separation‐of‐function dksA mutants reveal distinct roles of DksA in cSDR and in DNA repair

**Figure 11:**
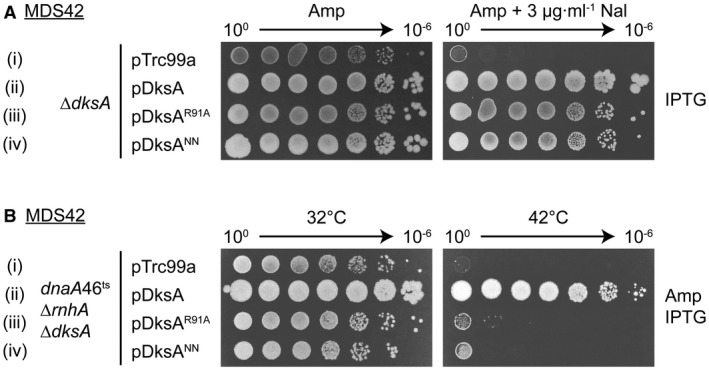
DksA coiled‐coil mutant proteins are able to support the repair of nalidixic acid‐induced DNA damage (A) but not cSDR (B). Wild‐type DksA protein and the DksA^R91A^ and DksA^NN^ mutants were expressed from pTrc99a plasmids using 1 mM IPTG. Strains used: (A) KM885, (B) KM882.

To ask if the roles of DksA in cSDR and DNA repair were identical, we tested several DksA mutations previously described as able to complement a *dksA* deletion. Fortuitously, two DksA point mutants, R91A and D71N/D74N (NN), displayed a separation‐of‐function phenotype. Both were able to complement the sensitivity of Δ*dksA* to Nal but neither suppressed the temperature sensitivity of *dnaA*46^ts^ Δ*rnhA* Δ*dksA*. This phenotype was seen in both MDS42 (Fig. 11) and MG1655 backgrounds (Fig. S4). The DksA R91A mutation lies in the coiled‐coil domain; DksA^NN^ carries two substitutions at the tip of the domain. Both DksA mutants are able to bind to RNAP, but are unable to inhibit transcription from the *rrnB* P1 promoter *in vivo* or *in vitro* (Parshin *et al.*, 2015). When overexpressed from a *lac* promoter, they can support the growth of Δ*dksA* on a minimal medium after prolonged incubation (Parshin *et al.*, 2015). These results suggest that the roles of DksA in cSDR and in the repair of Nal‐induced DSBs are not identical.

## Discussion

We report here a requirement of the *E. coli* RNAP‐associated protein, DksA, in the formation of new origins of replication (cSDR) in *dnaA*46^ts^ Δ*rnhA* or Δ*oriC* Δ*rnhA* mutants (Fig. 1). We also confirm and extend the observation that *dksA* mutants are sensitive to DNA damage induced by Nal (Meddows *et al.*, 2005). The requirement for DksA for both the formation of new origins and the repair of Nal‐induced DNA damage was demonstrated using a Δ*dksA* mutation or *rpoC* ^E677G^, an RNAP β′ subunit mutant that does not bind to DksA (Satory *et al.*, 2013; Ross *et al.*, 2016) (Figs 1, 2, 3).

DksA competes for access to the RNAP secondary channel with the anti‐backtracking factors GreA and GreB. An interplay between the three proteins within cells is complex, involving not only competition for RNAP, but also mutual control of the expression of their genes. Their effects on RNAP activity are in some instances redundant and in others competitive (Vinella *et al.*, 2012). Here, we show that DksA plays an active role both in cSDR and Nal‐induced DSB repair, rather than simply preventing the access of anti‐backtracking factors to the RNAP secondary channel. Thus, the requirement for DksA is not obviated by a *greA* deletion (Figs 7 and 8). This is in contrast to the mainly passive role of DksA in the repair of phleomycin‐induced DNA damage, which is attributed to the exclusion of GreA and thus to the enhancement of RNAP backtracking (Sivaramakrishnan *et al.*, 2017). The difference in the type of DNA damage inflicted by Nal versus phleomycin might account for this discrepancy. Phleomycin is a glycopeptide antibiotic that cleaves the DNA in the presence of metal cofactors and O_2_, leaving simple DSBs (Sleigh, 1976). In contrast, Nal‐induced DSBs carry 5′ type II topoisomerase adducts. Repair of such adducts in eukaryotic cells is known to involve different repair functions than simple DSBs (Aparicio *et al.*, 2016).

DksA, together with the RNAP β′ subunit, forms (p)ppGpp‐binding site 2, which is responsible for most of the effects of (p)ppGpp on transcription initiation (Ross *et al.*, 2016). It is conceivable, therefore, that it is not the lack of DksA *per se*, but the loss of the transcriptional control exerted by (p)ppGpp that is responsible for the inability of Δ*dksA *and *rpoC* ^E677G^ mutants to carry out cSDR and Nal‐induced DSB repair. Indeed, a lack of (p)ppGpp prevented cSDR in the *dnaA*46^ts^ Δ*rnhA* strain (Fig. 4B). However, the (p)ppGpp^0^ strain was only fractionally more sensitive to Nal than the wild type and more resistant than Δ*dksA* (Fig. 5C). These results suggest that the effect of Δ*dksA* on cSDR could be (p)ppGpp‐dependent. DksA plays an active, (p)ppGpp‐independent role in the repair of Nal‐induced DNA damage, consisting of DSBs and Top II DNA adducts. This lack of (p)ppGpp involvement is in contrast to the described role of (p)ppGpp in transcription‐coupled nucleotide excision repair (TC‐NER) (Kamarthapu *et al.*, 2016). However, the main role of (p)ppGpp in TC‐NER is to facilitate RNAP backtracking away from the damage, which allows efficient repair. In the case of Nal‐induced DNA damage, backtracking does not significantly enhance repair since the lack of GreA, an anti‐backtracking factor, did not rescue the sensitivity of *dksA* mutant (Fig. 8). A precedent for a (p)ppGpp‐independent role of DksA in genome stability exists, since, as previously reported, the suppression of replication–transcription clashes by DksA is likewise independent of (p)ppGpp (Tehranchi *et al.*, 2010).

An analysis of RNAP (p)ppGpp‐binding mutants did not fully clarify the importance of DksA–(p)ppGpp–RNAP interactions for cSDR and DNA repair. Surprisingly, the phenotype of the RNAP mutants was different than the phenotype of cells in the absence (p)ppGpp. Moreover, the two reactions (cSDR and DNA repair) displayed different (p)ppGpp effects. We found that mutations in the RNAP (p)ppGpp‐binding site 2 strongly inhibit cSDR in MDS42, but have less of an effect in the MG1655 background (Figs 4 and S1B). On the other hand, mutations in site 1, which is composed of the ω and β′ RNAP subunits, had little effect on MDS42 but inhibited growth in the MG1655 background. At present, we do not have an explanation for this phenotype. In contrast, mutations in site 2 enhanced the repair of DSBs, whereas site 1 mutations did not affect Nal sensitivity (Figs 5 and S2B). As mentioned above, site 2 accounts for most of the (p)ppGpp effects on transcription initiation (Ross *et al.*, 2016). The discrepancy between the phenotypes of site 2 mutants and ppGpp^0^ strain in cSDR and upon exposure to Nal was, therefore, unexpected. RNAP is not the only target of (p)ppGpp; perhaps the interaction of (p)ppGpp with other cellular components could explain the divergent phenotypes. However, the *in vivo* response of a RNAP sites 1 and 2 double mutant to nutritional shifts and amino acid starvation was equivalent to the (p)ppGpp^0^ strain, confirming that RNAP is the major target of (p)ppGpp (Ross *et al.*, 2016). The opposite effects of RNAP site 2 mutations on the ability to replicate via cSDR and repair Nal‐damaged DNA indicate that the two processes are not identical*.* Although DksA can bind to RNAP site 2 (Ross *et al.*, 2016), this interaction must be altered compared to the wild‐type RNAP.

Interestingly, two mutations in the coiled‐coil domain of DksA and the RNAP site 2 mutation displayed similar cSDR and DNA repair phenotypes. Both DksA^R91A^ and DksA^NN^, when overexpressed, supported the repair of Nal‐induced DNA damage in Δ*dksA*, but did not suppress the temperature sensitivity of the *dnaA*46^ts^ Δ*rnhA* Δ*dksA* strain (Fig. 11). The DksA residue R91 is positioned close to the RNAP (p)ppGpp‐binding site 2 residues β′ N680 and K681 and most likely forms salt bridges with the phosphate groups of (p)ppGpp (Molodtsov *et al.*, 2018). The DksA^R91A^ mutant protein binds to RNAP (albeit with reduced affinity) and similarly to β′^N680A K681A^ strongly inhibits (p)ppGpp‐dependent functions (Parshin *et al.*, 2015; Ross *et al.*, 2016). Thus, R91 is proposed to contribute to the formation of RNAP (p)ppGpp‐binding site 2. However, unlike the RNAP site 2 mutant, the DksA R91A substitution also limited DksA inhibition of transcription in the absence of (p)ppGpp (Ross *et al.*, 2016). The RNAP site 2 mutant and DksA^R91A^ both supported growth on minimal media after a prolonged incubation (Parshin *et al.*, 2015; Ross *et al.*, 2016). The DksA R91 residue interaction with the β′ rim helices may stabilize DksA in the secondary channel and aid in the positioning of the tip of the DksA coiled‐coil domain within the active center of RNAP (Parshin *et al.*, 2015). The DksA^NN^ mutant with D71N D74N substitutions at the tip of the coiled‐coil domain had similar phenotypes to DksA^R91A^, enabling DNA repair but not cSDR (Fig. 11). Residue D74 is very well conserved and was previously shown to be required for DksA function alone and together with (p)ppGpp at RNAP site 2 (Parshin *et al.*, 2015; Ross *et al.*, 2016). Residue D74 interacts with the substrate‐binding region of the RNAP active site and is essential for DksA activity (Parshin *et al.*, 2015). Taken together, these data suggest that the correct positioning of the DksA coiled‐coil tip in the RNAP active center is not required for the repair of Nal‐induced DNA damage but is critical for cSDR. Similarly, DksA^NN^ suppresses transcriptional pausing and transcription–replication conflicts even though it cannot regulate transcription initiation (Tehranchi *et al.*, 2010). This further supports the notion that the requirement for DksA in repair of Nal‐induced DNA damage involves its role in transcription elongation rather than transcription initiation.

DksA was dispensable for both DNA repair and cSDR in an RNAP mutant with a *rpoB* ^D444G^ substitution. D444 is located in a linker joining the βlobe/i4 domain and the main body of the β subunit (Fig. 10D), and could stabilize the transcription elongation complex (TEC). Several lines of evidence suggest that the *rpoB* ^D444G^ mutation destabilizes RNAP–DNA complexes during transcription initiation and/or elongation. The *rpoB* ^D444G^ mutation allows cells lacking accessory replicative helicases to overcome rich media synthetic lethality, enables their growth in the presence of an inverted *rrn* operon and facilitates replication restart (Baharoglu *et al.*, 2010). Similarly, the *rpoB* ^D444G^ mutation was also shown to enhance UV survival of *ruvABC* mutants**,** which are unable to resolve Holliday junctions, the last step of homologous recombination (Baharoglu *et al.*, 2010). It has been proposed that mutations that destabilize RNAP–DNA complexes facilitate the repair and the removal of obstacles that might otherwise block replication and create the need for RuvABC proteins to promote restart (Trautinger and Lloyd, 2002). In our study, the *rpoB* ^D444G^ mutation rescued the ability of *dksA* mutants to replicate *via* cSDR and repair Nal‐induced DNA damage (Fig. 10), which we also attribute to the decreased stability of TECs. A recent report demonstrated that DksA binding to RNAP in the absence of (p)ppGpp distorts both structures as compared to their apo‐forms or when bound in a ternary complex with (p)ppGpp (Molodtsov *et al.*, 2018). In the binary complex, the CT‐helix of DksA rotates the βlobe/i4 domain. The β^D444G^ substitution could, therefore, increase the flexibility of the βlobe/i4 domain, distorting the RNAP pincers, thus phenocopying DksA bound without (p)ppGpp in the secondary binding channel. We speculate that the destabilization of RNAP is required for both cSDR and Nal‐induced DNA repair.

Although no evidence for DksA destabilization of the TEC *in vitro *has been described (Roghanian *et al.*, 2015; Kamarthapu *et al.*, 2016), it is not ruled out that DksA might promote transcription termination *in vivo*. Indeed, DksA reduces transcription–replication clashes *in vivo*, implying that the protein acts on elongating RNAP (Tehranchi *et al.*, 2010). Note that we find that Rho, the transcription termination factor, is essential for recovery from Nal‐induced DNA damage (Fig. 9). Rho maintains genome stability by preventing replisome–TEC clashes that otherwise would induce replication fork arrest and DSBs (Washburn and Gottesman, 2011). *rho* and *dksA* mutations are not epistatic, suggesting that they affect different repair pathways, possibly interacting with different states of elongating RNAP.

cSDR and DNA repair presumably share the requirement for the removal of RNAP. For cSDR to occur, RNAP has to be removed to allow DNA Pol I access to the RNA primer. Rho factor removes both the RNAP and RNA:DNA hybrid and thus cannot support cSDR. We suggest that DksA might destabilize the elongating RNAP without unwinding the RNA:DNA hybrid. This notion requires that the 9–10 bp RNA:DNA hybrid in the TEC be sufficiently stable to persist after RNAP removal. Hybrids of this length have been purified (A. Mustaev, personal communication). Furthermore, *in vitro *construction of a TEC involves the addition of RNAP to an RNA:ssDNA hybrid. The hybrid is then further stabilized by the addition of the complementary DNA strand (Komissarova *et al.*, 2003). In cSDR, the RNA:DNA hybrid might be stabilized by RecA‐dependent formation of an R‐loop that would incorporate the 5′ end of the nascent transcript.

In the case of DNA repair, destabilization of the TEC by DksA could expose the DNA to allow the recombination and assembly of replication forks, as previously suggested (Meddows *et al.*, 2005). If DksA could remove RNAP without disturbing the RNA:DNA hybrid (and possibly the R‐loop upstream), DNA synthesis extending the RNA primer would allow the assembly of replication forks in a manner similar to cSDR. *In vitro* experiments supporting this notion have been reported. Thus, the *E. coli* replisome can use an RNA transcript as a primer to continue leading‐strand synthesis after a collision that displaces RNAP from the DNA template (Pomerantz and O'Donnell, 2008). Future experiments with reconstituted replication–transcription systems *in vitro* will be necessary to establish the precise role of DksA in cSDR and DNA repair.

## Experimental procedures

### Bacterial strains

All bacterial strains and plasmids used in this study are listed in Supplementary Tables 1 and 2. The strains used in supplementary figures and strains used for construction are in Supplementary Tables 3 and 4.

### Viability assays

*E. coli *strains were grown for 18 h at 37°C with shaking in LB broth. The cultures were then serially diluted 10‐fold in M9 salts. Five‐microliter aliquots were spotted on LB agar plates and incubated at 32°C and 42°C to assess the replication of *dnaA*46^ts^ strains *via* cSDR. To test nalidixic acid (Nal) sensitivity, 5 µl aliquots of 10‐fold dilutions were spotted on LB agar plates with and without Nal at a specified concentration. When required, 34 µg ml^–1^ of chloramphenicol or 100 µg ml^–1^ of ampicillin was added to the medium for plasmid maintenance. 1 mM IPTG was added to induce gene overexpression, where indicated. Nal sensitivity is presented as the percentage survival on LB + Nal vs. LB. All data points are shown on the graph with the mean marked in red and the standard deviation in black. Statistical analysis was performed using Kruskal–Wallis test with Dunn’s post test, comparing all the data sets. Alternatively, two sets of data were compared using the Mann–Whitney test. All experiments were performed at least twice; representative data sets are shown.

### Growth in the presence of Nal

Strains were grown overnight, diluted 100 µl into 5 ml LB in a 50 ml tube and grown at 37°C until cultures reached approx. 10^8^ cfu ml^–1^, which corresponds to OD_600 _~ 0.3–0.5, depending on the strain. Cultures were then diluted to OD_600_ = 0.01 in 10 ml of LB and split into two 50 ml tubes; one tube was treated with Nal to a final concentration of 3 µg ml^−1^. The cultures were then incubated, shaking, for 6 h at 37°C. Growth was monitored by measuring absorbance hourly. The viability of the cultures at 0, 3 and 6 h was assessed by serially diluting and spotting on LB plates in triplicates and calculating the cfu ml^−1^ after overnight incubation. The viability of each culture at *t*_0_ was arbitrarily set to 1 and the viability at *t*_3_ and *t*_6_ was normalized and presented graphically.

## Author contributions

The study was designed by KKM and MEG. KKM performed the experiments with the help of KK. KKM, RW and MEG analyzed and interpreted the data. KKM and MEG wrote the manuscript. The authors declare they have no conflict of interest with regard to this study.
