# Supplementary material for: DksA–RNA polymerase interactions support new origin formation and DNA repair in Escherichia coli
Source: Mol Microbiol. 2019 Mar 22;111(5):1382–97. doi: 10.1111/mmi.14227 (PMC6488371; doi:10.1111/mmi.14227)
Supplement: Supplementary file 1 [file MMI-111-1382-s001.pdf]

DksA-RNA polymerase interactions support new origin formation and DNA repair in *Escherichia coli*

Kamila K. Myka, Kira Küsters, Robert Washburn and Max E. Gottesman

SUPPLEMENTARY INFORMATION

- Supplementary experimental procedures
- Figures S1-S4
- Tables S1-S4
- Supplementary references

SUPPLEMENTARY EXPERIMENTAL PROCEDURES

Confirmation of ΔrelA and ppGpp⁰ phenotypes

Strains used for viability assays were also tested on M9 minimal agar with glucose with or without 1.6 mg ml^{-1} casamino acids or 1.6 µg ml^{-1} serine, methionine, glycine each (Ser-Met-Gly). 10 µl of a 10-fold dilution was spread on a section of a plate and incubated at 32°C if strains contained the *dnaA*46^{ts} mutation or 37°C for all other strains. Photographs were taken after 24h incubation. Δ*relA* strains display impaired growth on minimal agar with Ser-Met-Gly as compared with minimal agar without amino acids. Δ*relA* Δ*spoT* strains do not grow without casamino acids.

Phleomycin viability assay

E. coli strains were grown for 18 h at 37°C with shaking in LB broth. The cultures were then serially diluted ten-fold in M9 salts. 5 µl aliquots were spotted on LB agar plates with and without 1 µg ml^{-1} phleomycin and the plates were incubated at 37°C. Experiments were performed at least twice; representative datasets are shown.

Figure S1

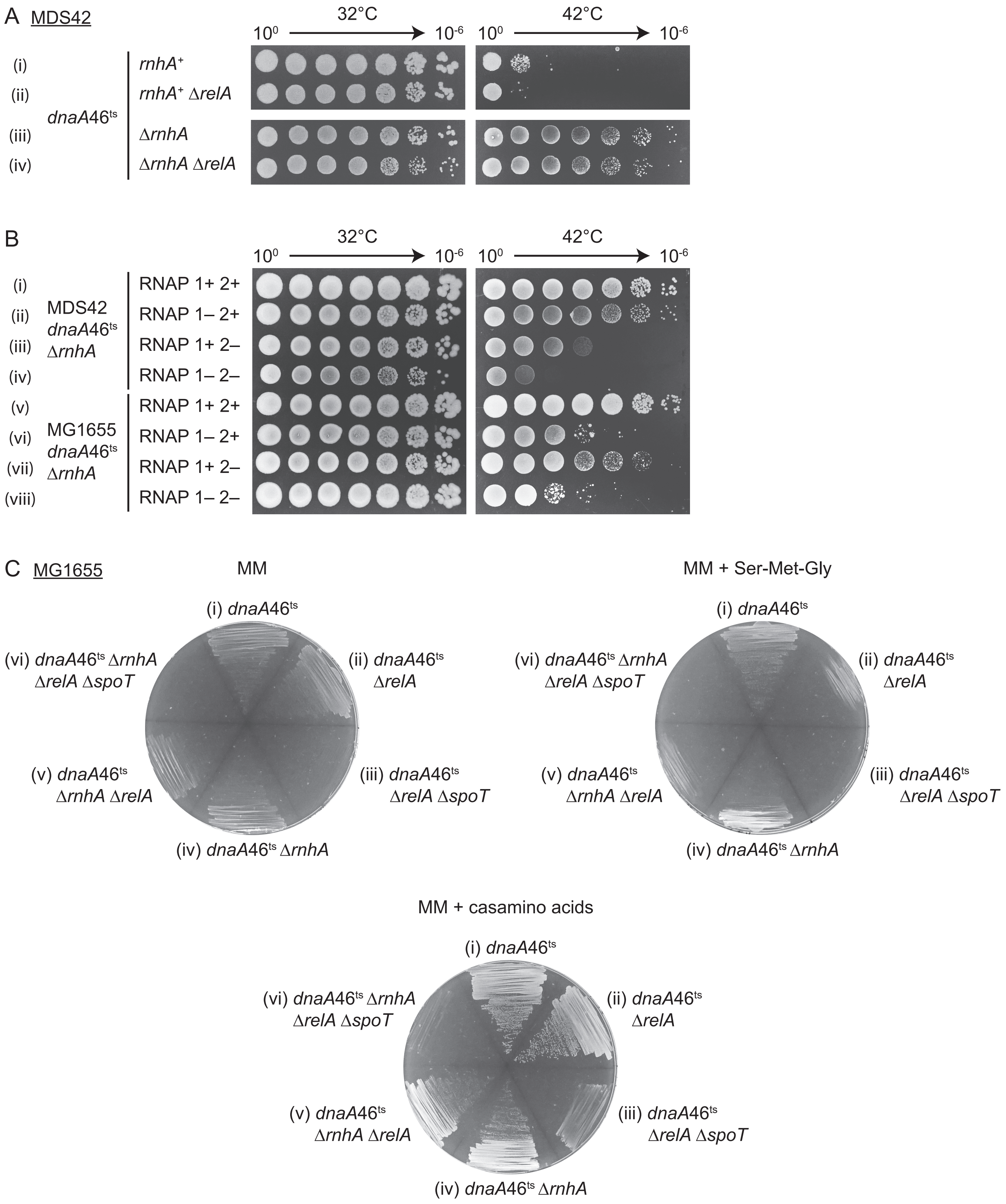

Fig. S1. The importance of (p)ppGpp for cSDR. Corresponds to Fig. 4. (A) Deletion of the gene encoding the major (p)ppGpp synthase, RelA, did not inhibit *oriC*-independent replication. Strains (i-iv): 10583, KM699, KM554, KM705. (B) RNAP (p)ppGpp binding site 2 plays a major role in cSDR in the MDS42 but not in the MG1655 background. cSDR is more impaired in RNAP (p)ppGpp binding site 1 mutants in MG1655-derived strains (i-viii): KK07B, KM901, KK09A, KM913, KM1120, KM1153, KM1165, KM1155. (C) Confirmation of Δ*relA* and ppGpp^0 phenotypes. Strains used are the same as in Fig. 4B (i-vi): KM712, KM1136, KM1137, KM1171, KM1173, KM1236.

Figure S2

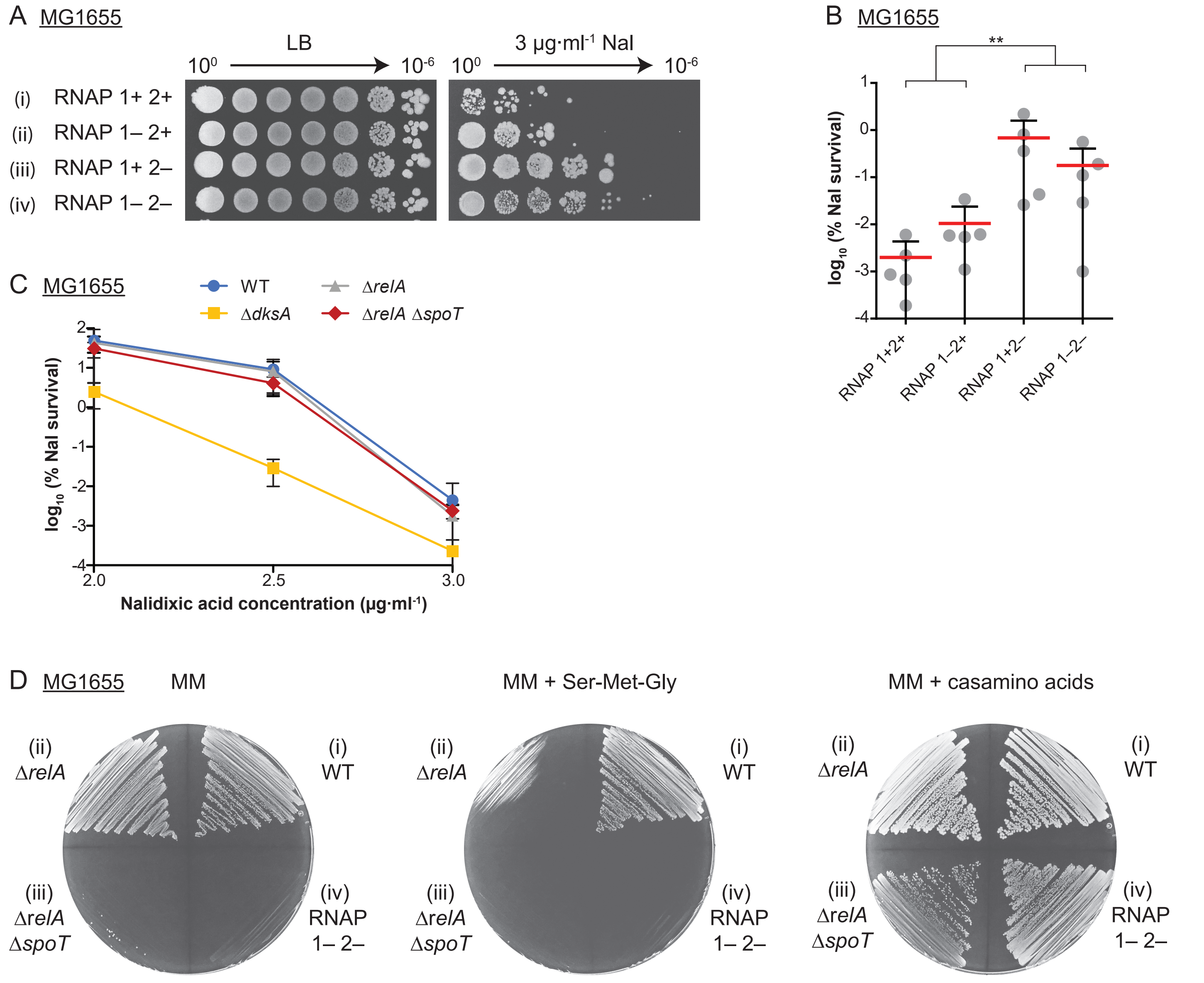

Fig. S2. RNAP (p)ppGpp-binding site 2 mutation enhances resistance to nalidixic acid. Corresponds to Fig. 5. (A) The RNAP site 1 mutation did not affect growth on Nal whereas the RNAP site 2 mutant was more resistant than wild-type. The Nal concentration used was 3 µg ml^{-1} instead of 4 µg ml^{-1} as in Fig. 5, because MG1655-derived strains are more sensitive to Nal than MDS42. Strains (i-iv): RLG14535, RLG14536, RLG14537, RLG14538. (B) Calculated percentage survival of strains on LB + Nal vs. LB alone. Graph shows mean percentage survival with one standard deviation. Statistical analysis was performed using a nonparametric two-tailed Mann-Whitney test, comparing combined data for RNAP 2+ vs. RNAP 2- mutants. **$p<0.01$, n=5. Standard deviation value higher than the mean value results in negative error bars crossing the x-axis when the y-axis is in logarithmic scale. (C) Calculated percentage survival of strains on LB + Nal vs. LB alone. Graph shows mean with standard deviation, for Nal concentration 2, 2.5, 3 µg ml^{-1}, n=6, 6, 7 for WT; n=4, 4, 3 for Δ*dksA* and n=4, 4, 5 for Δ*relA* and Δ*relA* Δ*spoT*. Strains as in Fig. 5C (i-iv): MG1655, KM773, RLG850, RLG847. (D) Confirmation of Δ*relA* and ppGpp^0 phenotypes. Strains used are the same as in Fig. 5C (i-iii): MG1655, RLG850, RLG847. Strain (iv) is RLG14538.

Figure S3

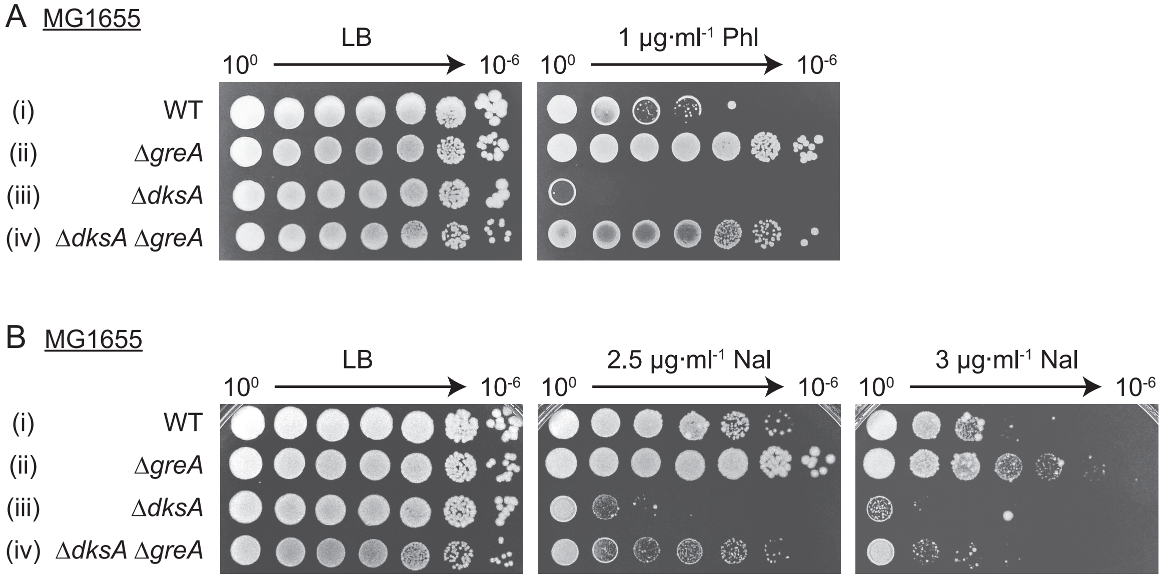

Fig. S3. Deletion of *greA* suppresses the sensitivity of a Δ*dksA* mutant to phleomycin (A) but not to nalidixic acid (B). Strains used are the same as in Fig. 8 (i-iv): MG1655, KM1034, KM773, KM1054.

Figure S4

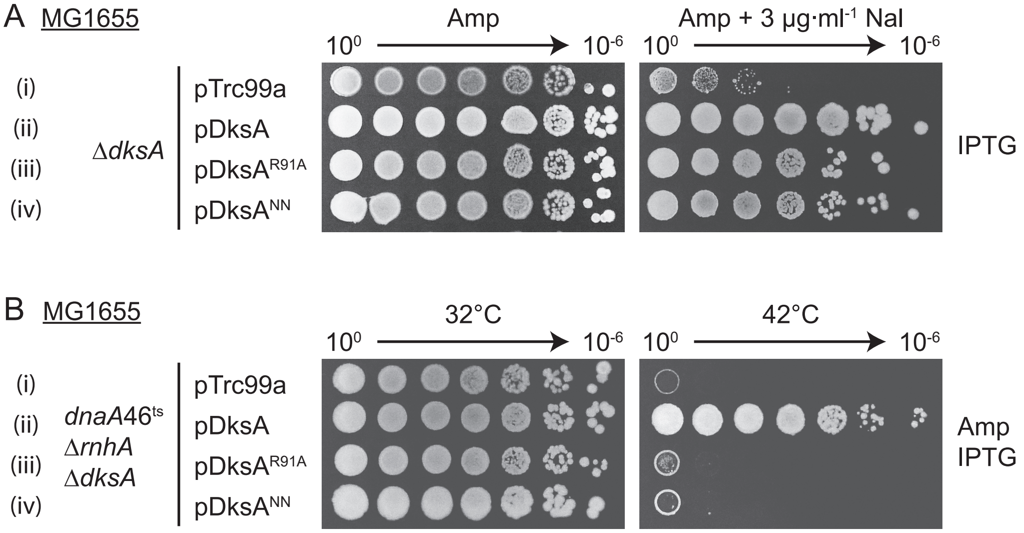

Fig. S4. DksA coiled-coil mutant proteins are able to support the repair of nalidixic acid-induced DNA damage (A) but not cSDR (B). As Fig. 11 but in the MG1655 background. Wild-type DksA protein and the $DksA^{R91A}$ and $DksA^{NN}$ mutants were expressed from a pTrc99a plasmids using 1 mM IPTG. Strains used: (A) KM773, (B) KM1064.

Table S1. Strains used in this study

Strain	Genotype	Construction / Source
10562	MDS42 Δ*rnhA*::cat	MDS42 x P1.PH379
10583	MDS42 *dnaA*46^{ts} *tna*::Tn*10*	MDS42 x P1.STL8297
10598	MDS42 *rho*15<amp>	MDS42 x P1.9993
12334	MDS42 *dnaA*46^{ts} *tna*::Tn*10* Δ*greA*::<kan>	10583 x P1.JW3148
12336	MDS42 *dnaA*46^{ts} *tna*::Tn*10* Δ*greB*::<kan>	10583 x P1.JW3369
12478	MDS42 Δ*dksA*::tet *rho*15<amp>	10598 x P1.11425
12481	MDS42 Δ*dksA*::<kan> *rpoB*^{D444G} *thiC*::Tn*10*	KM885 x P1.JJC4490
KK04A	MDS42 *rpoZ*^{WT}-kanR *rpoC*^{WT}-tetAR	KK01A x P1.RLG14535
KK05A	MDS42 *rpoZ*^{WT}-kanR *rpoC*$^{N680A\ K681A}$-tetAR	KK01A x P1.RLG14537
KK06A	MDS42 *dnaA*46^{ts} *tna*::Tn*10*(Tet^{s}) *rpoZ*^{WT}-kanR *rpoC*^{WT}-tetAR	KK02B x P1.RLG14535
KK07B	MDS42 *dnaA*46^{ts} *tna*::Tn*10*(Tet^{s}) Δ*rnhA*::cat *rpoZ*^{WT}-kanR *rpoC*^{WT}-tetAR	KK03A x P1.RLG14535
KK08A	MDS42 *dnaA*46^{ts} *tna*::Tn*10*(Tet^{s}) *rpoZ*^{WT}-kanR *rpoC*$^{N680A\ K681A}$-tetAR	KM869 x P1.RLG14535
KK09A	MDS42 *dnaA*46^{ts} *tna*::Tn*10*(Tet^{s}) Δ*rnhA*::cat *rpoZ*^{WT}-kanR *rpoC*$^{N680A\ K681A}$-tetAR	KM871 x P1.RLG14535
KM554	MDS42 *dnaA*46^{ts} *tna*::Tn*10* Δ*rnhA*::cat	10583 x P1.10562
KM586	MDS42 *dnaA*46^{ts} *tna*::Tn*10* Δ*rnhA*::cat Δ*greA*::<kan>	KM554 x P1.JW3148
KM588	MDS42 *dnaA*46^{ts} *tna*::Tn*10* Δ*rnhA*::cat Δ*greB*::<kan>	KM554 x P1.JW3369
KM644	MDS42 *dnaA*46^{ts} *tna*::Tn*10*(Tet^{s})	tetR removed from 10583 using chlortetacycline and fusaric acid[a]
KM650	MDS42 *dnaA*46^{ts} *tna*::Tn*10*(Tet^{s}) Δ*rnhA*::cat	tetR removed from KM554 using chlortetacycline and fusaric acid[a]
KM712	MG1655 *dnaA*46^{ts} *tna*::Tn*10*	MG1655 x P1.10583
KM727	MG1655 *dnaA*46^{ts} *tna*::Tn*10*(Tet^{s})	tetR removed from KM712 using chlortetacycline and fusaric acid[a]
KM773	MG1655 Δ*dksA*::<kan>	MG1655 x P1.JW0141
KM777	MDS42 Δ*rnhA*::cat Δ*dksA*::<kan>	10562 x P1.JW0141
KM801	MDS42 *dnaA*46^{ts} *tna*::Tn*10*(Tet^{s}) Δ*rnhA*::cat *rpoC*^{E677G} Δ*btuB*3191::Tn*10* Δ*yjeZ*::kan	KM650 x P1.CH2570
KM803	MDS42 *dnaA*46^{ts} *tna*::Tn*10*(Tet^{s}) Δ*dksA*::<kan>	KM644 x P1.JW0141
KM805	MDS42 *dnaA*46^{ts} *tna*::Tn*10*(Tet^{s}) Δ*dksA*::tet	KM644 x P1.11425
KM807	MDS42 *rpoC*^{E677G} Δ*btuB*3191::Tn*10* Δ*yjeZ*::kan	MDS42 x P1.CH2570
KM809	MDS42 *dnaA*46^{ts} *tna*::Tn*10*(Tet^{s}) *rpoC*^{E677G} Δ*btuB*3191::Tn*10* Δ*yjeZ*::kan	KM644 x P1.CH2570
KM829	MDS42 *rpoC*^{E677G} Δ*btuB*3191::Tn*10* Δ*yjeZ*::kan Δ*rnhA*::cat	KM807 x P1.10562
KM882	MDS42 *dnaA*46^{ts} *tna*::Tn*10*(Tet^{s}) Δ*rnhA*::cat Δ*dksA*::<kan>	KM650 x P1.JW0141
KM883	MDS42 *dnaA*46^{ts} *tna*::Tn*10*(Tet^{s}) Δ*rnhA*::cat Δ*dksA*::tet	KM650 x P1.11425
KM885	MDS42 Δ*dksA*::<kan>	MDS42 x P1.JW0141
KM899	MDS42 *dnaA*46^{ts} *tna*::Tn*10*(Tet^{s}) *rpoZ*$^{\Delta 2\text{-}5}$-kanR *rpoC*$^{R362A\ R417A\ K615A}$-tetAR	KM873 x P1.RLG14538
KM901	MDS42 *dnaA*46^{ts} *tna*::Tn*10*(Tet^{s}) Δ*rnhA*::cat *rpoZ*$^{\Delta 2\text{-}5}$-kanR *rpoC*$^{R362A\ R417A\ K615A}$-tetAR	KM875 x P1.RLG14538
KM911	MDS42 *dnaA*46^{ts} *tna*::Tn*10*(Tet^{s}) *rpoZ*$^{\Delta 2\text{-}5}$-kanR *rpoC*$^{R362A\ R417A\ K615A\ N680A\ K681A}$-tetAR	KM877 x P1.RLG14538
KM913	MDS42 *dnaA*46^{ts} *tna*::Tn*10*(Tet^{s}) Δ*rnhA*::cat *rpoZ*$^{\Delta 2\text{-}5}$-kanR *rpoC*$^{R362A\ R417A\ K615A\ N680A\ K681A}$-tetAR	KM879 x P1.RLG14538
KM915	MDS42 *rpoZ*$^{\Delta 2\text{-}5}$-kanR *rpoC*$^{R362A\ R417A\ K615A}$-tetAR	KM888 x P1.RLG14538
KM917	MDS42 *rpoZ*$^{\Delta 2\text{-}5}$-kanR *rpoC*$^{R362A\ R417A\ K615A\ N680A\ K681A}$-tetAR	KM890 x P1.RLG14538
KM982	MDS42 Δ*greA*::<> Δ*greB*::<kan>	KM980 x P1.JW3369
KM993	MG1655 *dnaA*46^{ts} *tna*::Tn*10*(Tet^{s}) Δ*rnhA*::cat	KM727 x P1.10562
KM1009	MDS42 *dnaA*46^{ts} *tna*::Tn*10*(Tet^{s}) *rpoB*^{D444G} *thiC*::Tn*10*	KM644 x P1.12481
KM1011	MDS42 *dnaA*46^{ts} *tna*::Tn*10*(Tet^{s}) Δ*rnhA*::cat *rpoB*^{D444G} *thiC*::Tn*10*	KM650 x P1.12481
KM1013	MDS42 *dnaA*46^{ts} *tna*::Tn*10*(Tet^{s}) *rpoB*^{D444G} *thiC*::Tn*10* Δ*dksA*::<kan>	KM1009 x P1.JW0141

Strain	Genotype	Construction / Source
KM1017	MDS42 *dnaA*46^{ts} *tna*::Tn*10*(Tet^{s}) Δ*rnhA*::cat *rpoB*^{D444G} *thiC*::Tn*10* Δ*dksA*::<kan>	KM1011 x P1.JW0141
KM1019	MDS42 *dnaA*46^{ts} *tna*::Tn*10* Δ*greA*::<> Δ*greB*::<kan>	KM982 x P1.10583
KM1021	MDS42 *dnaA*46^{ts} *tna*::Tn*10* Δ*rnhA*::cat Δ*greA*::<> Δ*greB*::<kan>	KM923 x P1.JW3369
KM1034	MG1655 Δ*greA*::<>	kanR removed from KM1027 using pCP20[b]
KM1047	MDS42 *rpoB*^{D444G} *thiC*::Tn*10*	MDS42 x P1.12481
KM1054	MG1655 Δ*greA*::<> Δ*dksA*::<kan>	KM1034 x P1.JW0141
KM1058	MG1655 *dnaA*46^{ts} *tna*::Tn*10*(Tet^{s}) Δ*greA*::<kan>	KM727 x P1.JW3148
KM1060	MG1655 *dnaA*46^{ts} *tna*::Tn*10*(Tet^{s}) Δ*dksA*::tet	KM727 x P1.11425
KM1062	MG1655 *dnaA*46^{ts} *tna*::Tn*10*(Tet^{s}) Δ*rnhA*::cat Δ*greA*::<kan>	KM993 x P1.JW3148
KM1064	MG1655 *dnaA*46^{ts} *tna*::Tn*10*(Tet^{s}) Δ*rnhA*::cat Δ*dksA*::tet	KM993 x P1.11425
KM1066	MG1655 *dnaA*46^{ts} *tna*::Tn*10*(Tet^{s}) Δ*greA*::<kan> Δ*dksA*::tet	KM1058 x P1.11425
KM1068	MG1655 *dnaA*46^{ts} *tna*::Tn*10*(Tet^{s}) Δ*rnhA*::cat Δ*greA*::<kan> Δ*dksA*::tet	KM1062 x P1.11425
KM1136	MG1655 *dnaA*46^{ts} *tna*::Tn*10* Δ*relA*251::kan	RLG850 x P1.10583
KM1137	MG1655 *dnaA*46^{ts} *tna*::Tn*10* Δ*relA*251::kan Δ*spoT*207::cat	RLG847 x P1.10583
KM1171	MG1655 *dnaA*46^{ts}:Tn*10* Δ*rnhA*::<>	KM716 x P1.10583
KM1173	MG1655 *dnaA*46^{ts} *tna*::Tn*10* Δ*rnhA*::<> Δ*relA*251::kan	KM1171 x P1.RLG847
KM1236	MG1655 *dnaA*46^{ts} *tna*::Tn*10* Δ*rnhA*::<> Δ*relA*251::kan Δ*spoT*207::cat	KM1173 x P1.RLG847
MDS42	MG1655 deleted for ~14% of the genome	(Posfai *et al.*, 2006)
MG1655	F- λ- *ilvG*- *rfb*-50 *rph*-1	R. Lloyd laboratory
RLG847	(= CF1693) MG1655 Δ*relA*251::kan Δ*spoT*207:cat	(Xiao *et al.*, 1991)
RLG850	(= CF1651) MG1655 Δ*relA*251::kan	(Metzger *et al.*, 1989)
RSW764	MDS42 Δ*rnhA*::cat Δ*oriC*::tet	recombineering into RSW763

[a] (Bochner *et al.*, 1980); [b] (Cherepanov and Wackernagel, 1995)

Table S2. Plasmids used in this study

Plasmid	Description	Source
pASKA	= pCA24N, $P_{T5\text{-}lac}$, *lacI*^{q}, Cm^{R}	(Kitagawa *et al.*, 2005)
pDksA(ASKA)	= pCA24N-JW0141 (with *dksA*)	(Kitagawa *et al.*, 2005)
pGreA(ASKA)	= pCA24N-JW3148 (with *greA*)	(Kitagawa *et al.*, 2005)
pGreB(ASKA)	= pCA24N-JW3369 (with *greB*)	(Kitagawa *et al.*, 2005)
pRLG6332	Complementation vector derived from pINIIIAI, P_{lpp} and P_{lac}, Amp^{R}	(Masui *et al.*, 1984)
pRLG6333	pRLG6332 with *dksA*	(Paul *et al.*, 2004)
pTrc99a	P_{trc} vector, *lacI*^{q}, pUC18 EcoRI-HindIII polylinker region, Amp^{R}	(Amann *et al.*, 1988)
pDksA	= pTRC-DksA-NPH, pTrc99a carrying *dksA* with N-terminal 6×His and PKA tags	(Parshin *et al.*, 2015)
pDksA^{R91}	= pTRC-AP53, pTRC-DksA-NPH with *dksA*^{R91A}	(Parshin *et al.*, 2015)
pDksA^{NN}	pTRC-DksA-NPH with *dksA*$^{D71N\ D74N}$	S. Borukhov laboratory

Table S3. Strains used in supplementary figures

Strain	Genotype	Construction / Source
KM699	MDS42 *dnaA*46^{ts} *tna*::Tn*10* Δ*relA*251::kan	10583 x P1.RLG850
KM705	MDS42 *dnaA*46^{ts} *tna*::Tn*10* Δ*rnhA*::cat Δ*relA*251::kan	KM554 x P1.RLG850
KM1120	MG1655 *dnaA*46^{ts} *tna*::Tn*10*(Tet^{s}) Δ*rnhA*::cat *rpoZ*^{WT}-kanR *rpoC*^{WT}-tetAR	KM1116 x P1.RLG14535
KM1153	MG1655 *dnaA*46^{ts} *tna*::Tn*10*(Tet^{s}) Δ*rnhA*::cat *rpoZ*$^{Δ2-5}$-kanR *rpoC*$^{R362A\ R417A\ K615A}$-tetAR	KM1149 x P1.RSW863
KM1155	MG1655 *dnaA*46^{ts} *tna*::Tn*10*(Tet^{s}) Δ*rnhA*::cat *rpoZ*$^{Δ2-5}$-kanR *rpoC*$^{R362A\ R417A\ K615A\ N680A\ K681A}$-tetAR	KM1151 x P1.RSW863

Strain	Genotype	Construction / Source
KM1165	MG1655 *dnaA*46^{ts} *tna*::Tn*10*(Tet^{s}) Δ*rnhA*::cat *rpoZ*^{WT}-kanR *rpoC*$^{N680A\ K681A}$-tetAR	KM1126 x P1.RSW863
RLG14535	MG1655 *rpoZ*^{WT}-kanR *rpoC*^{WT}-tetAR (1+2+)	(Ross *et al.*, 2016)
RLG14536	MG1655 *rpoZ*$^{\Delta 2\text{-}5}$-kanR *rpoC*$^{R362A\ R417A\ K615A}$-tetAR (1-2+)	(Ross *et al.*, 2016)
RLG14537	MG1655 *rpoZ*^{WT}-kanR *rpoC*$^{N680A\ K681A}$-tetAR (1+2-)	(Ross *et al.*, 2016)
RLG14538	MG1655 *rpoZ*$^{\Delta 2\text{-}5}$-kanR *rpoC*$^{R362A\ R417A\ K615A\ N680A\ K681A}$-tetAR (1-2-)	(Ross *et al.*, 2016)

Table S4. Strains used for construction

Strain	Genotype	Construction / Source
9993	HME57 *rho*15<amp>	D. Court laboratory
11105	MDS42 Δ*greA*::<kan>	MDS42 x P1.JW3148
11425	MDS42 *dksA*::tet	MDS42 x P1.RLG7238
CH2570	*lacZ*-U118 Δ*yjaZ*::*kan* Δ*btuB*3191::*Tn10* *rpoC*^{E677G}	(Satory *et al.*, 2013)
JJC4490	JJC40 *rpoB*^{D444G} *thiC*::Tn*10*	(Baharoglu *et al.*, 2010)
JW0141	F- Δ*(araD-araB)*567 Δ*dksA*761*::kan* Δ*lacZ*4787(::rrnB-3) λ^{-} *rph*-1 Δ*(rhaD-rhaB)*568 *hsdR*514	(Baba *et al.*, 2006)
JW0204	F- Δ*(araD-araB)*567 Δ*rnhA*733*::kan* Δ*lacZ*4787(::rrnB-3) λ^{-} *rph*-1 Δ*(rhaD-rhaB)*568 *hsdR*514	(Baba *et al.*, 2006)
JW3148	F- Δ*(araD-araB)*567 Δ*lacZ*4787(::rrnB-3) λ^{-} Δ*greA*788*::kan* *rph*-1 Δ*(rhaD-rhaB)*568 *hsdR*514	(Baba *et al.*, 2006)
JW3369	F- Δ*(araD-araB)*567 Δ*lacZ*4787(::rrnB-3) λ^{-} Δ*greB*740*::kan* *rph*-1 Δ*(rhaD-rhaB)*568 *hsdR*514	(Baba *et al.*, 2006)
KK01A	MDS42 *rpoZ*^{WT}-kanR	MDS42 x P1.RLG14535
KK02B	MDS42 *dnaA*46^{ts} *tna*::Tn*10*(Tet^{s}) *rpoZ*^{WT}-kanR	KM644 x P1.RLG14535
KK03A	MDS42 *dnaA*46^{ts} *tna*::Tn*10*(Tet^{s}) Δ*rnhA*::cat *rpoZ*^{WT}-kanR	KM650 x P1.RLG14535
KM716	MG1655 Δ*rnhA*::<>	kanR removed from RSW899 using pCP20[b]
KM869	MDS42 *dnaA*46^{ts} *tna*::Tn*10*(Tet^{s}) *rpoC*$^{N680A\ K681A}$-tetAR	KM644 x P1.RLG14537
KM871	MDS42 *dnaA*46^{ts} *tna*::Tn*10*(Tet^{s}) Δ*rnhA*::cat *rpoC*$^{N680A\ K681A}$-tetAR	KM650 x P1.RLG14537
KM873	MDS42 *dnaA*46^{ts} *tna*::Tn*10*(Tet^{s}) *rpoC*$^{R362A\ R417A\ K615A}$-tetAR	KM644 x P1.RLG14536
KM875	MDS42 *dnaA*46^{ts} *tna*::Tn*10*(Tet^{s}) Δ*rnhA*::cat *rpoC*$^{R362A\ R417A\ K615A}$-tetAR	KM650 x P1.RLG14536
KM877	MDS42 *dnaA*46^{ts} *tna*::Tn*10*(Tet^{s}) *rpoC*$^{R362A\ R417A\ K615A\ N680A\ K681A}$-tetAR	KM644 x P1.RLG14538
KM879	MDS42 *dnaA*46^{ts} *tna*::Tn*10*(Tet^{s}) Δ*rnhA*::cat *rpoC*$^{R362A\ R417A\ K615A\ N680A\ K681A}$-tetAR	KM650 x P1.RLG14538
KM888	MDS42 *rpoC*$^{R362A\ R417A\ K615A}$-tetAR	MDS42 x P1.RLG14536
KM890	MDS42 *rpoC*$^{R362A\ R417A\ K615A\ N680A\ K681A}$-tetAR	MDS42 x P1.RLG14538
KM923	MDS42 *dnaA*46^{ts} *tna*::Tn*10* Δ*rnhA*::cat Δ*greA*::<>	kanR removed from KM586 using pCP20[b]
KM980	MDS42 Δ*greA*::<>	kanR removed from 11105 using pCP20[b]
KM1027	MG1655 Δ*greA*::<kan>	MG1655 x P1.JW3148
KM1114	MG1655 *dnaA*46^{ts} *tna*::Tn*10*(Tet^{s}) *rpoZ*^{WT}-kanR	KM727 x P1.RLG14535
KM1116	MG1655 *dnaA*46^{ts} *tna*::Tn*10*(Tet^{s}) Δ*rnhA*::cat *rpoZ*^{WT}-kanR	KM993 x P1.RLG14535
KM1122	MG1655 *dnaA*46^{ts} *tna*::Tn*10*(Tet^{s}) *rpoZ*$^{\Delta 2\text{-}5}$-kanR	KM727 x P1.RLG14536
KM1126	MG1655 *dnaA*46^{ts} *tna*::Tn*10*(Tet^{s}) *rpoZ*^{WT}-kanR *rpoC*$^{N680A\ K681A}$-tetAR	KM1114 x P1.RLG14537
KM1149	MG1655 *dnaA*46^{ts} *tna*::Tn*10*(Tet^{s}) *rpoZ*$^{\Delta 2\text{-}5}$-kanR *rpoC*$^{R362A\ R417A\ K615A}$-tetAR	KM1122 x P1.RLG14536
KM1151	MG1655 *dnaA*46^{ts} *tna*::Tn*10*(Tet^{s}) *rpoZ*$^{\Delta 2\text{-}5}$-kanR *rpoC*$^{R362A\ R417A\ K615A\ N680A\ K681A}$-tetAR	KM1122 x P1.RLG14538
PH379	RFM430 *rnhA*::cat	(Usongo *et al.*, 2008)
RLG7238	RLG5950 *dksA*::tet	(Rutherford *et al.*, 2007)
RSW763	MDS42 Δ*rnhA*::cat pSIM6	MDS42 pSIM6 x P1.PH379
RSW863	MG1655 Δ*rnhA*::cat	MG1655 x P1.PH379

Strain	Genotype	Construction / Source
RSW899	MG1655 Δ*rnhA*::<kan>	MG1655 x P1.JW0204
STL8297	*dnaA*46^{ts} *tna*::Tn*10*	(Foti *et al.*, 2005)

[b] (Cherepanov and Wackernagel, 1995)

SUPPLEMENTARY REFERENCES

Amann, E., Ochs, B. and Abel, K.J., (1988) Tightly regulated *tac* promoter vectors useful for the expression of unfused and fused proteins in *Escherichia coli*. *Gene* **69**: 301-315.

Baba, T., Ara, T., Hasegawa, M., Takai, Y., Okumura, Y., Baba, M., Datsenko, K.A., Tomita, M., Wanner, B.L. and Mori, H., (2006) Construction of *Escherichia coli* K-12 in-frame, single-gene knockout mutants: the Keio collection. *Mol Syst Biol* **2**: 2006 0008.

Baharoglu, Z., Lestini, R., Duigou, S. and Michel, B., (2010) RNA polymerase mutations that facilitate replication progression in the *rep uvrD recF* mutant lacking two accessory replicative helicases. *Mol Microbiol* **77**: 324-336.

Bochner, B.R., Huang, H.C., Schieven, G.L. and Ames, B.N., (1980) Positive selection for loss of tetracycline resistance. *J Bacteriol* **143**: 926-933.

Cherepanov, P.P. and Wackernagel, W., (1995) Gene disruption in *Escherichia coli*: Tc^R and Km^R cassettes with the option of Flp-catalyzed excision of the antibiotic-resistance determinant. *Gene* **158**: 9-14.

Foti, J.J., Schienda, J., Sutera, V.A., Jr. and Lovett, S.T., (2005) A bacterial G protein-mediated response to replication arrest. *Mol Cell* **17**: 549-560.

Kitagawa, M., Ara, T., Arifuzzaman, M., Ioka-Nakamichi, T., Inamoto, E., Toyonaga, H. and Mori, H., (2005) Complete set of ORF clones of *Escherichia coli* ASKA library (*A* complete *S*et of *E. coli K*-12 ORF *A*rchive): unique resources for biological research. *DNA Res* **12**: 291-299.

Masui, Y., Mizuno, T. and Nouye, M., (1984) Novel high-level expression cloning vehicles: 10^4-fold amplification of *Escherichia coli* minor protein. *Bio-Technol* **2**: 81-85.

Metzger, S., Schreiber, G., Aizenman, E., Cashel, M. and Glaser, G., (1989) Characterization of the *relA*1 mutation and a comparison of *relA*1 with new *relA* null alleles in *Escherichia coli*. *J Biol Chem* **264**: 21146-21152.

Parshin, A., Shiver, A.L., Lee, J., Ozerova, M., Schneidman-Duhovny, D., Gross, C.A. and Borukhov, S., (2015) DksA regulates RNA polymerase in *Escherichia coli* through a network of interactions in the secondary channel that includes Sequence Insertion 1. *Proc Natl Acad Sci U S A* **112**: E6862-6871.

Paul, B.J., Barker, M.M., Ross, W., Schneider, D.A., Webb, C., Foster, J.W. and Gourse, R.L., (2004) DksA: a critical component of the transcription initiation machinery that potentiates the regulation of rRNA promoters by ppGpp and the initiating NTP. *Cell* **118**: 311-322.

Posfai, G., Plunkett, G., 3rd, Feher, T., Frisch, D., Keil, G.M., Umenhoffer, K., Kolisnychenko, V., Stahl, B., Sharma, S.S., de Arruda, M., Burland, V., Harcum, S.W. and Blattner, F.R., (2006) Emergent properties of reduced-genome *Escherichia coli*. *Science* **312**: 1044-1046.

Ross, W., Sanchez-Vazquez, P., Chen, A.Y., Lee, J.H., Burgos, H.L. and Gourse, R.L., (2016) ppGpp binding to a site at the RNAP-DksA interface accounts for its dramatic effects on transcription initiation during the stringent response. *Mol Cell* **62**: 811-823.

Rutherford, S.T., Lemke, J.J., Vrentas, C.E., Gaal, T., Ross, W. and Gourse, R.L., (2007) Effects of DksA, GreA, and GreB on transcription initiation: insights into the mechanisms of factors that bind in the secondary channel of RNA polymerase. *J Mol Biol* **366**: 1243-1257.

Satory, D., Halliday, J.A., Sivaramakrishnan, P., Lua, R.C. and Herman, C., (2013) Characterization of a novel RNA polymerase mutant that alters DksA activity. *J Bacteriol* **195**: 4187-4194.

Usongo, V., Nolent, F., Sanscartier, P., Tanguay, C., Broccoli, S., Baaklini, I., Drlica, K. and Drolet, M., (2008) Depletion of RNase HI activity in *Escherichia coli* lacking DNA topoisomerase I leads to defects in DNA supercoiling and segregation. *Mol Microbiol* **69**: 968-981.

Xiao, H., Kalman, M., Ikehara, K., Zemel, S., Glaser, G. and Cashel, M., (1991) Residual guanosine 3',5'-bispyrophosphate synthetic activity of *relA* null mutants can be eliminated by *spoT* null mutations. *J Biol Chem* **266**: 5980-5990.
